# Water-Reaching Platform for Longitudinal Assessment of Cortical Activity and Fine Motor Coordination Defects in a Huntington Disease Mouse Model

**DOI:** 10.1523/ENEURO.0452-22.2022

**Published:** 2023-01-06

**Authors:** Yundi Wang, Marja D. Sepers, Dongsheng Xiao, Lynn A. Raymond, Timothy H. Murphy

**Affiliations:** 1 Department of Psychiatry, Kinsmen Laboratory of Neurological Research, Detwiller Pavilion, University of British Columbia, Vancouver, British Columbia V6T 1Z3, Canada; 2 Djavad Mowafaghian Centre for Brain Health, 2215 Wesbrook Mall, University of British Columbia, Vancouver, British Columbia V6T 1Z3, Canada

**Keywords:** calcium imaging, cortex, Huntington disease, mesoscale, mouse, water-reaching

## Abstract

Huntington disease (HD), caused by dominantly inherited expansions of a CAG repeat results in characteristic motor dysfunction. Although gross motor defects have been extensively characterized in multiple HD mouse models using tasks such as rotarod and beam walking, less is known about forelimb deficits. We develop a high-throughput alternating reward/nonreward water-reaching task and training protocol conducted daily over approximately two months to simultaneously monitor forelimb impairment and mesoscale cortical changes in GCaMP activity, comparing female zQ175 (HD) and wild-type (WT) littermate mice, starting at ∼5.5 months. Behavioral analysis of the water-reaching task reveals that HD mice, despite learning the water-reaching task as proficiently as wild-type mice, take longer to learn the alternating event sequence as evident by impulsive (noncued) reaches and initially display reduced cortical activity associated with successful reaches. At this age gross motor defects determined by tapered beam assessment were not apparent. Although wild-type mice displayed no significant changes in cortical activity and reaching trajectory throughout the testing period, HD mice exhibited an increase in cortical activity, especially in the secondary motor and retrosplenial cortices, over time, as well as longer and more variable reaching trajectories by approximately seven months. HD mice also experienced a progressive reduction in successful performance. Tapered beam and rotarod tests as well as reduced DARPP-32 expression (striatal medium spiny neuron marker) after water-reaching assessment confirmed HD pathology. The water-reaching task can be used to inform on a daily basis, HD and other movement disorder onset and manifestation, therapeutic intervention windows, and test drug efficacy.

## Significance Statement

The movement disorder, Huntington disease (HD), has been extensively studied in preclinical settings using mouse models of disease examining gross motor and balance defects. Little however, is known regarding forelimb deficits and underlying cortical circuit changes. Using a high-throughput alternating reward/nonreward water-reaching task, we characterized early event sequence defects as evident by impulsive reaches and reduced cortical activity associated with successful reaches in HD mice aged ∼5.5 months. Progressive forelimb movement defects first become apparent at ∼6.5 months of age with corresponding increases in cortical activity associated with reaching observed over time. The water-reaching task can be used to inform on a daily basis HD disease onset and progression, therapeutic intervention windows and test drug efficacy.

## Introduction

Synaptic and circuit changes that precede progressive striatal medium spiny neuron (MSN) and cortical neuronal loss in Huntington disease (HD) result in a characteristic triad of clinical manifestations, including: motor dysfunction, cognitive impairment, and neuropsychiatric disorders (McColgan and Tabrizi, 2018; Cepeda and Levine, 2022). The hallmark motor symptoms of HD include fine motor incoordination, chorea, bradykinesia, rigidity and difficulties with balance and gait.

Since the discovery that dominantly inherited expansions >39 of a CAG triplet repeat in exon 1 of the *HTT* gene causes HD (MacDonald, 1993), over 50 distinct mouse and rat models with increasingly better face and construct validity have been developed (Pouladi et al., 2013; Menalled et al., 2014). Although several assessments such as rotarod, balance beam and gait tasks (such as the footprint test) are commonly used to assess motor defects in HD mice (Pallier et al., 2009; Brooks et al., 2012; Abada et al., 2013; Southwell et al., 2016), bodyweight remains a major confound of these tasks (McFadyen et al., 2003; Batka et al., 2014), necessitating the development and usage of other behavioral assessments.

In clinical settings, impairments in reaching and/or skilled hand movements have been observed in HD patients (Klein et al., 2011). Skilled forelimb movement learning and performance has also been examined in HD mice using automated home-cage lever pulling systems (Woodard et al., 2017, 2021), demonstrating that HD motor learning deficits are related to impaired striatal neuronal plasticity (Woodard et al., 2021). Given reaching toward a target and manipulating objects is commonly used in our daily lives and the general “reach-to-grasp” features of forelimb movements has been shown to be similar between humans and rodents (Galiñanes et al., 2018), preclinical behavioral assessment of skilled forelimb reaching tasks could improve our understanding of HD movement defects. Water-reaching tasks further enable longitudinal multi-trial assessment providing a high throughput system for behavioral phenotyping throughout the duration of disease progression, and may detect more subtle motor learning and movement defects. Combining water-reaching assessment with simultaneous recording of widefield cortical activity further enables examination of pathophysiological circuit changes underlying HD movement defects.

To date, few studies exist examining these wide-scale cortical circuit changes in HD mouse models. Using 3D magnetic resonance imaging, arteriolar cerebral blood volume level changes in the striatum and motor cortex were observed in HD mice beginning at three months of age which worsened overtime (Liu et al., 2021). Hemodynamic measurements are, however, indirect indicators of neuronal activity. Using mesoscale voltage-sensitive dye imaging, our group has shown that hindlimb stimulation evokes a larger area and longer lasting cortical response in anesthetized HD compared with wild-type (WT) mice (Sepers et al., 2021). Given that recent neurophysiology studies have demonstrated the involvement of multiple brain regions in sensation, cognition, and movement (Pinto et al., 2019; Steinmetz et al., 2019), widefield functional assessment of cortical circuit changes during task performance can provide longitudinal read-outs of HD pathology across brain networks. Accordingly, we used a water-reaching task to demonstrate progressive changes in widefield cortical activity and skilled forelimb movement defects. The full-length *HTT* knock-in heterozygous zQ175 mouse model was employed because of its greater construct validity over other HD mouse models (Pouladi et al., 2013).

## Materials and Methods

### Animals

All experiments and procedures were conducted in accordance with the Canadian Council on Animal Care and approved by the University of British Columbia Committee on Animal Care (protocols A18-0036 and A19-0076). Mice were group housed with two to four mice per cage under a controlled 12/12 h light/dark cycle (7 A.M. lights on, 7 P.M. lights off). Standard laboratory mouse diet was available *ad libitum*. Water was available *ad libitum* except during the duration of head-fixed water-reaching behavioral testing and when mice were tapered back to *ad libitum* water consumption. Surgery and subsequent behavioral testing was performed on 6 female heterozygous zQ175 knock-in C57BL/6 mice expressing GCaMP6s and six female wild-type (WT) littermates as controls starting at approximately five months of age. zQ175 C57BL/6 mice and WT littermates, both expressing GCaMP6s, were obtained by first crossing heterozygous zQ175 C57BL/6 mice (https://www.jax.org/strain/029928) with homozygous transgenic Thy-1 GCaMP6s line 4.3 C57BL/6 mice (Howard Hughes Medical Institute Janelia Research Campus; Dana et al., 2014; Sofroniew et al., 2016) then crossing subsequent offspring to obtain homozygous GCaMP6s expression. Animal tissue was collected through ear clipping at weaning. DNA extraction and PCR analysis were subsequently used to determine genotype. Health status and weight of all animals was assessed daily. A limitation of our study is that only female mice were employed. Learning and task noncompliance in water reaching (Galiñanes et al., 2018) has been reported in WT mice. Female mice were selected since mice performing the task (that were originally from different cages) could be co-housed.

### Animal surgery

All mice were subjected to head-bar and chronic transcranial window surgery as previously described (Murphy et al., 2016; Silasi et al., 2016) and allowed to recover for a week before commencement of behavioral testing. Briefly, an incision and skin retraction over the cortex was made enabling a glass coverslip to be applied using Metabond clear dental cement (Parkell; Product: C&B Metabond) onto unthinned bone. A steel head-fixation bar was also placed 4 mm posterior between bregma and the bar edge.

### Behavioral testing timeline

During the handling period, mice were first habituated to daily human contact for three weeks. Mice then underwent surgery and were allowed to recover for one week before initial tapered beam assessment (5 d). After 2 d of initial tapered beam testing, mice were also habituated to first the confinement tube only, then to the confinement tube and head fixation and, finally the confinement tube, head fixation and experimental setup. The duration of head fixation was progressively increased at a rate of ∼7 min/d for 5 d.

Mice were then water restricted for skilled forelimb head-fixed water-reaching behavioral training and testing. From the water-reaching task, mice had the potential to receive ∼1 ml/d of water. Given variation in weight because of *ad libitum* food consumption and excrement, mice who either performed poorly or lost >0.5 g in weight compared with the previous day were given up to ∼1.1 ml of task-independent water. All mice received ∼100 μl of additional water daily. As such, all mice received ∼1.1 ml of task-independent and/or behavioral test-derived water daily. The humane endpoint was defined as a maximal weight loss of 15% from a prewater-restricted baseline weight. No mice reached the humane endpoint during the duration of the study.

After at least 60 d (maximum 67 d) of water restriction and water-reaching behavioral training and testing, mice were tapered back to *ad libitum* access to water. During this readjustment period, mice received 1.1 ml of task-independent water on the first day. In subsequent days mice received progressively increased water at a rate of ∼500 μl/d for ∼4 d. This additional water was administered at three different times during the light cycle to prevent water intoxication. After stabilization of mouse weight, *ad libitum* access to water was restored for the duration of the behavioral testing.

Accelerating rotarod (4 d) and final tapered beam testing (7 d) was then conducted with a 1-d recovery period between the two behavior assessments. All animals were killed with intraperitoneal injection of pentobarbital sodium (240 mg/kg) and transcardially perfused with first 10-ml PBS then 10 ml 10% neutral buffered formalin (NBF). Whole brains were removed and postfixed in NBF for postmortem immunohistochemistry.

### Head-fixed water-reaching test

Mice underwent head-bar and chronic transcranial surgery and were trained to reach for water under head-fixed conditions following in part a previously described protocol (Galiñanes et al., 2018). All mice were water restricted after habituation to the confinement tube, head-fixation and experimental set up. A platform which extends 1.5 cm from the base of the confinement tube allowed the mice to rest their forepaws while not reaching for water. The water spout was fashioned using a blunted 22G needle bent at a 90° angle. The starting position of the water spout was ∼0.75–1 cm posterior from the tip of the snout and positioned laterally so that the water drop made minimal contact with the whiskers. At this position, mice could touch the water spout with their paws and feel the water drop if they groomed which transitioned to reaching. For mice which did not groom, the water drop was allowed to touch their whisker pad which promoted grooming and transition into reaching toward the spout. Once mice started reaching, the distance of the water spout was gradually increased until a final distance of ∼1.5 cm lateral and ∼0.5 cm posterior to the tip of the snout was achieved. Only mice that reached the final distance with a success rate of at least ∼80% on day 15 were included for further analysis. Although all 12 mice underwent the entire behavioral testing timeline, two WT mice did not engage to the extent and/or meet these predetermined criteria and were not included in subsequent water-reaching analysis.

Trial structure included alternating reward (water drop presented) and no reward (no water drop, despite identical presentation of visual and auditory cues) trials. Unless an electronic failure occurred, all trials started with a rewarded trial. The experimental setup was illuminated with infrared LED illuminator lights. A Raspberry Pi single-board computer and custom Python script was used to control camera recording, blue and green light used to illuminate the cortex, cue light signal, cue buzzer signal, water solenoid to deliver the water reward and capacitive touch sensor connected to the water spout.

At the start of reward and no reward trials, a Raspberry Pi infrared night vision camera (320 × 320 pixels; 60 Hz) and a 1M60 Pantera CCD camera (Dalsa) enabled behavioral and GCaMP activity recording, respectively. The cortex was illuminated using alternating green and blue light providing information about hemodynamic changes and exciting GCaMP, respectively and collected as 12-bit images through the Dalsa camera using XCAP imaging software (120 Hz). Binning camera pixels (8 × 8) produced a resolution of 68 μm/pixel. These imaging parameters have been used previously for widefield cortical GCaMP imaging (Vanni and Murphy, 2014; Xiao et al., 2017).

A 0.1-s duration green LED light flash 2 s after the start of camera recording was followed by a 0.1-s buzzer tone in the case of nonrewarded trials or a buzzer tone combined with simultaneous ∼20-μl water reward in the case of rewarded trials 6 s after the start of camera recording. For rewarded trials, if a spout touch was detected by the capacitive touch sensor (Adafruit Industries) after delivery of the water reward, the Picamera and Dalsa camera recording would cease 4 s after the time of spout touch. If a touch was not detected or it was a nonrewarded trial, camera recording would cease 10 s after delivery of the reward. Rewarded trials therefore ranged from 10–16 s and nonrewarded trials were 16 s in length. The intertrial interval (ITI) was 4 s. A total of ∼120 trials over a duration of ∼39 min were conducted daily for 60–67 d.

Since the capacitive touch sensor was found to not accurately determine reaches, touches and/or contact with the water spout, all trials were manually blind scored with six categories for rewarded trials (disregard trial, no reach, groom, success, partial fail and complete fail) and three categories for nonrewarded trials (disregard trial, no reach and unrewarded reach). Trials which were disregarded included trials where there was either an electronic failure (e.g., water solenoid delivered too much or too little water, truncated behavioral and/or cortical activity imaging video was recorded, etc.), experimenter intervention (e.g., during training, after the animal would cause the position of the water spout to move because of vigorous grooming/reaching, etc.), or when the trial was deemed too difficult to score. No reach trials referred to those wherein the mouse did not groom or lift either both or one paw off of the resting platform in a forward reaching motion. Groom trials referred to the mouse engaging in grooming behavior. Complete fail trials consisted of the mouse reaching forward but being unable to make contact with or obtain the water drop. Partial fail trials consisted of the mouse reaching forward and making contact with the water drop but then being unable to bring the water to its mouth to drink. No rewarded trials scored with the category “unrewarded reach” referred to either the animal engaging in grooming behavior or reaching behavior even when no water was present on the water spout. Grooming behavior was included in this category to reduce scoring subjectivity between natural grooming behavior and groom-to-reach behavior. Mice were also observed to switch between grooming and reaching the spout. Successful trials on day 8 were also scored for the absence and presence of early reaches defined as reaches that occurred before water reward presentation. Early reaches and unrewarded reaches are referred to as impulsive reaches and reflect an event sequence defect.

### Accelerating rotarod test

Mice were tested as previously described (Woodard et al., 2021). Briefly, mice were tested for four consecutive days on the rotarod (Ugo Basille) accelerated from 5 to 40 RPM over a total time period of 300 s. Mice received three trials per day with a 2-h intertrial interval (ITI). A fall was defined as the mouse falling from the rotarod or completing a rotation holding onto the rod and not trying to right itself at any point during the rotation. If a fall or full rotation occurred, the trial was ended and the time recorded. Mice that reached the maximum allowed time were scored as 300 s, and the trial ended. The average latency to fall for the three trials was scored.

### Tapered beam test

Mice were tested using an automated touch sensing tapered beam test (Ardesch et al., 2017). Briefly, conductive paint surfaces serving as input electrodes to four 12-channel capacitive touch sensors (Adafruit Industries) connected to a Raspberry Pi single-board computer recorded the start and finish times to traverse the beam using a custom Python script. The beam measured 100 cm in length tapering from 3.5 to 0.5 cm with a wider 1-cm base component extending to the left and right 1 cm below the upper surface of the beam. Mice received four trials per day for five and seven consecutive days during the first and second round of tapered beam testing. Average time required to traverse the beam across the four trials was scored.

### Immunohistochemistry

Coronal brain sections were cut on a vibratome at 50-μm thickness (Leica VT1000S, Leica Microsystems GmbH). Slides were then boiled in sodium citrate (10 mm sodium citrate, 0.05% Tween 20, pH 6) to allow antigen retrieval. After washing, slices were permeabilized with 0.3% Triton X-100, blocked with BlockAid Blocking Solution (Invitrogen) and Image-iT FX Signal Enhancer (Invitrogen) before 1:100 primary antibody labeling overnight [rabbit monoclonal anti-DARPP-32 (Abcam; ab40801) and mouse anti-NeuN (MilliporeSigma; ZMS377)]. PBS, 0.1% Tween 20 (PBST) washing was then followed by 1:1000 secondary antibody labeling [rhodamine (TRITC) AffiniPure goat anti-mouse IgG(H+L) (JacksonImmuno Research Laboratories Inc.; 115-025-003) and AlexaFluorTM647-R-phycoerythtin goat anti-rabbit IgG(H+L) (A20991, Thermo Fisher Scientific)]. Sections were washed with PBST and mounted on glass coverslips with Prolong Gold Antifade Mountant (Thermo Fisher Scientific; P36930) for subsequent imaging. Sections were imaged with a 10× and 63× objective using an up-right Leica imaging system (SP8 DIVE). Staining intensity was determined using ImageJ software. Relative intensity values are expressed relative to background.

### Kinematic and mesoscale GCaMP analysis

To accommodate for varying rewarded trial lengths, the first 10 s were examined for all rewarded and nonrewarded trials.

#### Kinematic analysis of forelimb skilled reaching behavior

Deeplabcut as described previously (Mathis et al., 2018) was used to track body parts (right and left forepaws and mouth) and equipment landmarks of interest (platform and spout). Subsequent analyses were conducted using a custom MATLAB code. For Figure 3 and Extended Data Figure 3-1, the distance from the height of the platform to the height of the spout was calculated and represents the distance to the spout (spout distance). The Euclidean distance of the left paw trajectories was calculated from the time of water reward delivery to 1.1 s afterward for all successful rewarded trials. This time period was selected since it corresponded to the time needed to complete a successful reach. Euclidean distances traveled by the left paw were then binned. Histogram bin size reflects multiples of spout distance. For example, bin four contains successful rewarded trials where the Euclidean distances of the left paw trajectories were 4× that of the spout distance. Euclidean distances are reported as multiples of spout distance since this distance represents the most efficient route the left paw could take to reach the spout. The average Euclidean distance and SD were calculated for each mouse then genotype averaged. For Figure 6 the *y*-direction traces of left paw movement for each trial are depicted.

#### GCaMP image processing and analysis

All GCaMP image processing and analysis were conducted using custom MATLAB codes. All GCaMP responses were movement and hemodynamic artifact corrected by subtracting changes in green reflectance signals from observed green epi-fluorescence (Vanni et al., 2017) and expressed as percentages relative to baseline responses (F–F_0_/F_0_)*100 where F_0_ is the baseline from the start of the trial to water reward delivery. For region-based analysis, the brain-to-atlas approach in MesoNet (Xiao et al., 2021) was used to register cortical images to a common atlas using predicted cortical landmarks to determine regions of interest (ROIs). A 5 × 5 pixel region centered in each ROI was used for examination of peak amplitude and baseline SD. Peak amplitude was calculated from the baseline (defined as 1–5 s from the start of the trial) to the peak. Cortical area activated was determined as pixel intensities >4× SD of the baseline (1–5 s). Contralateral (right) and ipsilateral (left) hemisphere ROIs include the primary motor (M1), secondary motor (M2), somatosensory mouth (sspm), somatosensory forelimb (sspfl), somatosensory hindlimb (ssphl), somatosensory area unassigned (sspun), somatosensory nose (sspn), somatosensory barrel field domain (sspbfd), somatosensory trunk (ssptr), primary visual (visp), retrosplenial lateral agranular part (rspagl), and retrosplenial dorsal (rspd) cortices. Forelimb movements after presentation of the water drop in rewarded trials were classified as successful or failed reaches. As such, subsequent ΔF/F analyses for these trial types were concentrated during the time period after the water drop (until 4 s afterward). However, reaching during nonrewarded trials could occur throughout the trial and was not necessarily associated with a cue. Therefore, each ROI’s peak amplitude for unrewarded reach trials was evaluated across the entire trial duration (total 10 s).

### Experimental design, statistical analysis, and code accessibility

All experimenters were blinded during the analysis. Unless otherwise stated, two-way ANOVA and Šídák’s multiple comparisons *post hoc* test were used (Table 1). Statistical analysis was calculated using GraphPad Prism. Alpha level for all tests was *p* = 0.05. The code used for the analysis is available from the corresponding authors on request.

**Table 1:** Statistical table of all analyses

	Data structure	Type of test	Test values and power
Figure 2*A*	All groups normally distributed	Two-way ANOVA, mixed effects model with Šídák’s multiple comparisons *post hoc* tests	Day: *F*_(11,95)_ = 28.02; *p* < 0.0001 Genotype: *F*_(1,95)_ = 9.878; *p* = 0.0022 Interaction: *F*_(11,95)_ = 7.477; *p* < 0.0001
Figure 2*B*	All groups normally distributed	Two-way ANOVA, mixed effects model with Šídák’s multiple comparisons *post hoc* tests	Day: *F*_(3.717,29.40)_ = 4.793; *p* = 0.0050 Genotype: *F*_(1,8)_ = 4.694; *p* = 0.0621 Interaction: *F*_(11,87)_ = 7.020; *p* < 0.0001
Figure 2*C*	All groups normally distributed	Two-way ANOVA, mixed effects model with Šídák’s multiple comparisons *post hoc* tests	Day: *F*_(11,95)_ = 44.93; *p* < 0.0001 Genotype: *F*_(1,95)_ = 0.004561; *p* = 0.9463 Interaction: *F*_(11,95)_ = 5.818; *p* < 0.0001
Figure 2*D*	All groups normally distributed	Two-way ANOVA, mixed effects model with Šídák’s multiple comparisons *post hoc* tests	Day: *F*_(10,79)_ = 6.369; *p* <0.0001 Genotype: *F*_(1,8)_ = 3.430; *p* = 0.1012 Interaction: *F*_(10,79)_ = 1.966; *p* = 0.0484
Figure 3*D*	All groups normally distributed	Two-way ANOVA with Šídák’s multiple comparisons *post hoc* tests	Day: *F*_(1,8)_ = 0.1888; *p* = 0.6754 Genotype: *F*_(1,8)_ = 4.469; *p* = 0.0475 Interaction: *F*_(1,8)_ = 2.922; *p* = 0.1257
Figure 3*E*	All groups normally distributed	Two-way ANOVA with Šídák’s multiple comparisons *post hoc* tests	Day: *F*_(1,8)_ = 0.0003947; *p* = 0.9846 Genotype: *F*_(1,8)_ = 8.939; *p* = 0.0173 Interaction: *F*_(1,8)_ = 0.7136; *p* = 0.4228
Figure 5*B*	All groups normally distributed	Two-way ANOVA with Šídák’s multiple comparisons *post hoc* tests	ROI: *F*_(2.943,23.54)_ = 44.67; *p* < 0.0001 Genotype: *F*_(1,8)_ = 5.925; *p* = 0.0409 Interaction: *F*_(11,88)_ = 0.7083; *p* = 0.7276
Figure 5*C*	All groups normally distributed	Two-way ANOVA with Šídák’s multiple comparisons *post hoc* tests	ROI: *F*_(2.896,23.17)_ = 37.34; *p* < 0.0001 Genotype: *F*_(1,8)_ = 5.967; *p* = 0.0404 Interaction: *F*_(11,88)_ = 2.204; *p* = 0.0210
Figure 5*D*	All groups normally distributed	Two-way ANOVA	ROI: *F*_(11,72)_ = 18.57; *p* < 0.0001 Day: *F*_(1,72)_ = 0.005939; *p* = 0.9388 Interaction: *F*_(11,72)_ = 0.5529; *p* = 0.8600
Figure 5*E*	All groups normally distributed	Two-way ANOVA with Šídák’s multiple comparisons *post hoc* tests	ROI: *F*_(11,110)_ = 58.60; *p* < 0.0001 Day: *F*_(1,10)_ = 5.521; *p* = 0.0407 Interaction: *F*_(11,110)_ = 2.665; *p* = 0.0046
Figure 5*G*	All groups normally distributed	Two-way ANOVA with Šídák’s multiple comparisons *post hoc* tests	Day: *F*_(3,24)_ = 0.4308; *p* = 0.7328 Genotype: *F*_(1,8)_ = 1.763; *p* = 0.2209 Interaction: *F*_(3,24)_ = 3.286; *p* = 0.0380 *p = 0.0455 (Fig. 5F, M2)
Extended Data Figure 5-1*A*	All groups normally distributed	Two-way ANOVA	ROI: *F*_(3.281,19.69)_ = 27.01; *p* < 0.0001 Day: *F*_(1,6)_ = 0.02610; *p* = 0.8770 Interaction: *F*_(11,66)_ = 0.4249; *p* = 0.9397
Extended Data Figure 5-1*B*	All groups normally distributed	Two-way ANOVA	ROI: *F*_(11,110)_ = 42.14; *p* < 0.0001 Day: *F*_(1,10)_ = 0.7094; *p* = 0.4193 Interaction: *F*_(11,110)_ = 1.220; *p* = 0.2825
Extended Data Figure 5-2, M1	All groups normally distributed	Two-way ANOVA	Day: *F*_(3,24)_ = 0.6755; *p* = 0.5755 Genotype: *F*_(1,8)_ = 0.4845; *p* = 0.5061 Interaction: *F*_(3,24)_ = 1.978; *p* = 0.1442
Extended Data Figure 5-2, M2	All groups normally distributed	Two-way ANOVA with Šídák’s multiple comparisons *post hoc* tests	Day: *F*_(3,24)_ = 0.4308; *p* = 0.7328 Genotype: *F*_(1,8)_ = 1.763; *p* = 0.2209 Interaction: *F*_(3,24)_ = 3.286; *p* = 0.0380
Extended Data Figure 5-2, sspm	All groups normally distributed	Two-way ANOVA with Šídák’s multiple comparisons *post hoc* tests	Day: *F*_(3,24)_ = 2.408; *p* = 0.0920 Genotype: *F*_(1,8)_ = 9.237; *p* = 0.0161 Interaction: *F*_(3,24)_ = 1.961; *p* = 0.1468
Extended Data Figure 5-2, sspfl	All groups normally distributed	Two-way ANOVA	Day: *F*_(3,24)_ = 1.600; *p* = 0.2155 Genotype: *F*_(1,8)_ = 1.585; *p* = 0.2435 Interaction: *F*_(3,24)_ = 2.394; *p* = 0.0934
Extended Data Figure 5-2, ssphl	All groups normally distributed	Two-way ANOVA	Day: *F*_(3,24)_ = 1.104; *p* = 0.3667 Genotype: *F*_(1,8)_ = 1.654; *p* = 0.2344 Interaction: *F*_(3,24)_ = 0.7276; *p* = 0.5456
Extended Data Figure 5-2, sspun	All groups normally distributed	Two-way ANOVA	Day: *F*_(3,24)_ = 1.111; *p* = 0.3649 Genotype: *F*_(1,8)_ = 3.723; *p* = 0.0898 Interaction: *F*_(3,24)_ = 0.400; *p* = 0.7542
Extended Data Figure 5-2, sspn	All groups normally distributed	Two-way ANOVA	Day: *F*_(3,24)_ = 3.104; *p* = 0.0455 Genotype: *F*_(1,8)_ = 3.916; *p* = 0.0832 Interaction: *F*_(3,24)_ = 0.6181; *p* = 0.6101
Extended Data Figure 5-2, sspbfd	All groups normally distributed	Two-way ANOVA with Šídák’s multiple comparisons *post hoc* tests	Day: *F*_(3,24)_ = 0.803; *p* = 0.5050 Genotype: *F*_(1,8)_ = 6.088; *p* = 0.0389 Interaction: *F*_(3,24)_ = 1.759; *p* = 0.1831
Extended Data Figure 5-2, ssptr	All groups normally distributed	Two-way ANOVA	Day: *F*_(3,24)_ = 0.8346; *p* = 0.4881 Genotype: *F*_(1,8)_ = 0.5758; *p* = 0.4697 Interaction: *F*_(3,24)_ = 2.098; *p* = 0.1271
Extended Data Figure 5-2, visp	All groups normally distributed	Two-way ANOVA with Šídák’s multiple comparisons *post hoc* tests	Day: *F*_(3,24)_ = 0.05820; *p* = 0.9811 Genotype: *F*_(1,8)_ = 4.586; *p* = 0.646 Interaction: *F*_(3,24)_ = 3.481; *p* = 0.0322
Extended Data Figure 5-2, rspagl	All groups normally distributed	Two-way ANOVA	Day: *F*_(3,24)_ = 1.253; *p* = 0.3126 Genotype: *F*_(1,8)_ = 0.0001239; *p* = 0.9914 Interaction: *F*_(3,24)_ = 1.322; *p* = 0.2903
Extended Data Figure 5-2, rspd	All groups normally distributed	Two-way ANOVA	Day: *F*_(3,24)_ = 2.364; *p* = 0.0962 Genotype: *F*_(1,8)_ = 4.888; *p* = 0.0580 Interaction: *F*_(3,24)_ = 0.5185; *p* = 0.6735
Extended Data Figure 5-3, M1	All groups normally distributed	Two-way ANOVA	Day: *F*_(3,24)_ = 1.481; *p* = 0.2448 Genotype: *F*_(1,8)_ = 0.8359; *p* = 0.3873 Interaction: *F*_(3,24)_ = 1.899; *p* = 0.1568
Extended Data Figure 5-3, M2	All groups normally distributed	Two-way ANOVA with Šídák’s multiple comparisons *post hoc* tests	Day: *F*_(3,24)_ = 0.3644; *p* = 0.7793 Genotype: *F*_(1,8)_ = 16.91; *p* = 0.0034 Interaction: *F*_(3,24)_ = 0.6271; *p* = 0.6046
Extended Data Figure 5-3, sspm	All groups normally distributed	Two-way ANOVA	Day: *F*_(3,24)_ = 0.5004; *p* = 0.6855 Genotype: *F*_(1,8)_ = 1.391; *p* = 0.2721 Interaction: *F*_(3,24)_ = 0.3365; *p* = 0.7991
Extended Data Figure 5-3, sspfl	All groups normally distributed	Two-way ANOVA	Day: *F*_(3,24)_ = 0.4592; *p* = 0.7133 Genotype: *F*_(1,8)_ = 1.079; *p* = 0.3293 Interaction: *F*_(3,24)_ = 0.2794; *p* = 0.8397
Extended Data Figure 5-3, ssphl	All groups normally distributed	Two-way ANOVA	Day: *F*_(3,24)_ = 0.8751; *p* = 0.4678 Genotype: *F*_(1,8)_ = 0.2996; *p* = 0.5991 Interaction: *F*_(3,24)_ = 0.6056; *p* = 0.6178
Extended Data Figure 5-3, sspun	All groups normally distributed	Two-way ANOVA	Day: *F*_(3,24)_ = 0.4799; *p* = 0.6994 Genotype: *F*_(1,8)_ = 0.2840; *p* = 0.6086 Interaction: *F*_(3,24)_ = 0.6877; *p* = 0.5688
Extended Data Figure 5-3, sspn	All groups normally distributed	Two-way ANOVA	Day: *F*_(3,24)_ = 0.7687; *p* = 0.5228 Genotype: *F*_(1,8)_ = 0.5080; *p* = 0.4962 Interaction: *F*_(3,24)_ = 0.4713; *p* = 0.7051
Extended Data Figure 5-3, sspbfd	All groups normally distributed	Two-way ANOVA	Day: *F*_(3,24)_ = 1.244; *p* = 0.3158 Genotype: *F*_(1,8)_ = 3.942; *p* = 0.0824 Interaction: *F*_(3,24)_ = 1.321; *p* = 0.2906
Extended Data Figure 5-3, ssptr	All groups normally distributed	Two-way ANOVA with Šídák’s multiple comparisons *post hoc* tests	Day: *F*_(3,24)_ = 0.7592; *p* = 0.5280 Genotype: *F*_(1,8)_ = 5.924; *p* = 0.0410 Interaction: *F*_(3,24)_ = 1.043; *p* = 0.3914
Extended Data Figure 5-3, visp	All groups normally distributed	Two-way ANOVA with Šídák’s multiple comparisons *post hoc* tests	Day: *F*_(3,24)_ = 0.5061; *p* = 0.6818 Genotype: *F*_(1,8)_ = 5.549; *p* = 0.0463 Interaction: *F*_(3,24)_ = 0.5052; *p* = 0.6824
Extended Data Figure 5-3, rspagl	All groups normally distributed	Two-way ANOVA with Šídák’s multiple comparisons *post hoc* tests	Day: *F*_(3,24)_ = 0.9880; *p* = 0.4151 Genotype: *F*_(1,8)_ = 17.03; *p* = 0.0033 Interaction: *F*_(3,24)_ = 1.097; *p* = 0.3695
Extended Data Figure 5-3, rspd	All groups normally distributed	Two-way ANOVA with Šídák’s multiple comparisons *post hoc* tests	Day: *F*_(3,24)_ = 0.7315; *p* = 0.5434 Genotype: *F*_(1,8)_ = 19.47; *p* = 0.0022 Interaction: *F*_(3,24)_ = 0.3894; *p* = 0.7617
Figure 6*B*	All groups normally distributed	Unpaired *t* test	*t*_(8)_ = 2.937; *p* = 0.0188
Figure 6*C*	All groups normally distributed	Two-way ANOVA with Šídák’s multiple comparisons *post hoc* tests	ROI: *F*_(2.925,29.25)_ = 81.47; *p* < 0.0001 Genotype: *F*_(1,10)_ = 7.783; *p* = 0.0191 Interaction: *F*_(11,110)_ = 0.6211; *p* = 0.8075
Figure 6*D*	All groups normally distributed	Two-way ANOVA	ROI: *F*_(3.194,31.94)_ = 36.54; *p* < 0.0001 Genotype: *F*_(1,10)_ = 1.531; *p* = 0.2442 Interaction: *F*_(11,110)_ = 1.762; *p* = 0.0693
Extended Data Figure 6-1*A*	All groups normally distributed	Two-way ANOVA	ROI: *F*_(11,110)_ = 56.08; *p* < 0.0001 Genotype: *F*_(1,10)_ = 2.540; *p* = 0.1421 Interaction: *F*_(11,110)_ = 1.060; *p* = 0.3998
Extended Data Figure 6-1*B*	All groups normally distributed	Two-way ANOVA	ROI: *F*_(11,110)_ = 44.86; *p* < 0.0001 Genotype: *F*_(1,10)_ = 0.2573; *p* = 0.6230 Interaction: *F*_(11,110)_ = 0.6316; *p* = 0.7984
Extended Data Figure 6-1*C*, area activated after water reward	All groups normally distributed	Unpaired *t* test	*t*_(10)_ = 1.214; *p* = 0.2527
Figure 7*A*	All groups normally distributed	Two-way ANOVA with Šídák’s multiple comparisons *post hoc* tests	Day: F_(4,40)_ = 9.970; *p* < 0.0001 Genotype: *F*_(1,10)_ = 2.743; *p* = 0.1287 Interaction: F_(4,10)_ = 3.294; *p* = 0.0200
Figure 7*B*	All groups normally distributed	Two-way ANOVA, mixed effects model with Šídák’s multiple comparisons *post hoc* tests	Day: F_(6,59)_ = 8.636; *p* < 0.0001 Genotype: *F*_(1,10)_ = 15.21; *p* = 0.0030 Interaction: F_(6,59)_ = 0.9735; *p* = 0.4512
Figure 7*C*	All groups normally distributed	Two-way ANOVA with Šídák’s multiple comparisons *post hoc* tests	Age: *F*_(1,10)_ = 8.251; *p* = 0.0166 Genotype: *F*_(1,10)_ = 5.446; *p* = 0.0418 Interaction: *F*_(1,10)_ = 8.372; *p* = 0.0160
Figure 7*D*	All groups normally distributed	Two-way ANOVA with Šídák’s multiple comparisons *post hoc* tests	Day: *F*_(3,10)_ = 13.52; *p* < 0.0001 Genotype: *F*_(1,10)_ = 27.94; *p* = 0.0004 Interaction: *F*_(3,30)_ = 1.098; *p* = 0.3653
Figure 7*G*, DARPP-32	All groups normally distributed	Unpaired *t* test	*t*_(6)_ = 9.765; *p* < 0.0001
Figure 7*G*, NeuN	All groups normally distributed	Unpaired *t* test	*t*_(6)_ = 0.5644; *p* = 0.5930

## Results

### Overview of experimental assessment timeline

**Figure 1.**
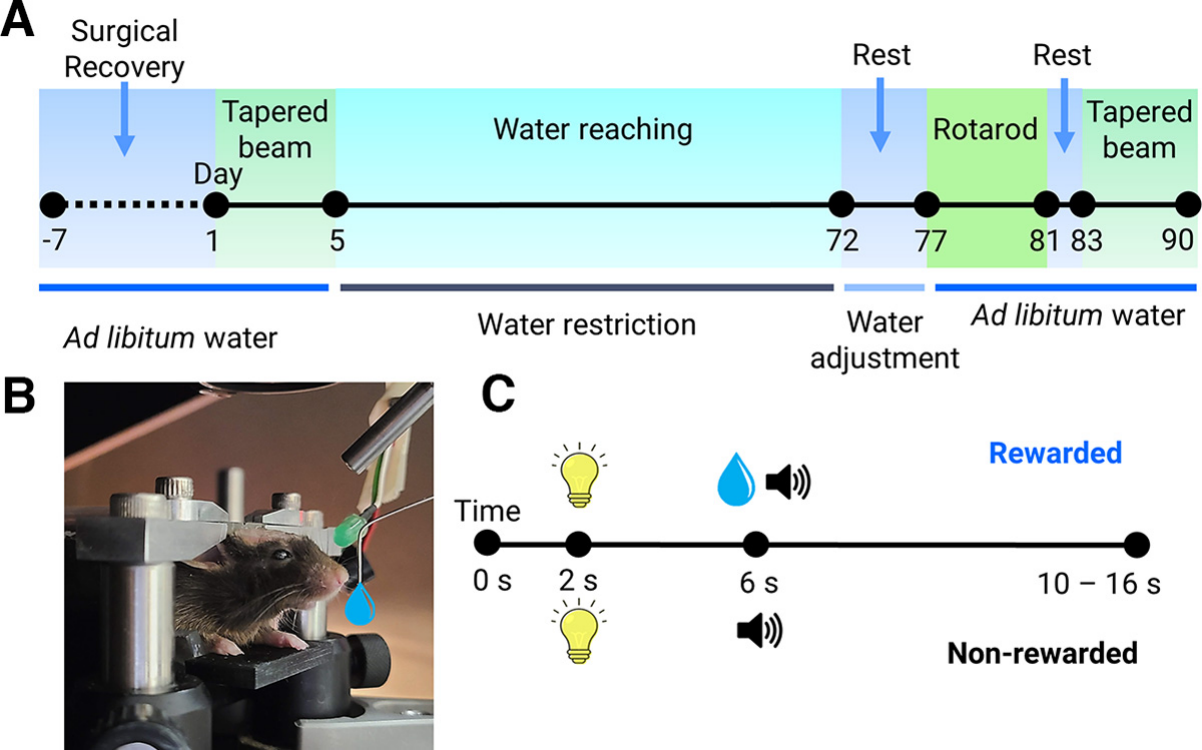
Scheme of behavioral testing. ***A***, Animals were allowed to recover from surgery (∼1 week) before initial tapered beam testing (5 d) which was followed by water-restricted forelimb water-reaching testing (60–67 d). Mice were tapered back to *ad libitum* water consumption (∼5 d) before rotarod testing (4 d) and final tapered beam testing (7 d) with 1 d in between rotarod and tapered beam testing to allow stamina recovery. ***B***, Side-view image of a representative head-fixed mouse in the water-reaching task. ***C***, Trial structure for the water-reaching task where alternating rewarded and nonrewarded trials were performed. The intertrial interval was 4 s. A visual cue 2 s after initiation of camera recording was followed by an auditory cue and water drop reward for rewarded trials and only an auditory cue for nonrewarded trials both 6 s after initiation of camera recording. If a spout touch was detected after water reward delivery, the rewarded trial ended 4 s after the spout touch was detected. In cases where a spout touch was not detected, the rewarded trial timed out 10 s after water reward delivery. All nonrewarded trials ended 10 s after the auditory cue. Total trial length was therefore 16 s for nonrewarded trials and could range from 10 to 16 s for rewarded trials depending on if a water spout touch was detected. For consistency, all trial lengths were truncated to 10 s in total for subsequent analyses.

The behavioral testing timeline is depicted in Figure 1*A*. After approximately one week of chronic window and head-fixation bar surgical recovery, all mice underwent tapered beam training and testing to examine baseline gross motor function. Mice were then water restricted and trained to perform skilled forelimb water-reaching (Fig. 1*B*). Behavioral camera recording combined with markerless pose estimation (Mathis et al., 2018) enabled tracking and assessment of progressive forelimb coordination defects. Simultaneous recording of cortical activity using GCaMP6 mesoscale imaging further enabled assessment of progressive cortical circuit changes. After the completion of water-reaching assessment, mice were allowed to rest and were tapered back to *ad libitum* water consumption. Mice then underwent rotarod testing and a second round of tapered beam testing to examine HD gross motor defects. Finally, HD pathology was determined using immunohistochemistry staining for DARPP-32, a striatal medium spiny neuron (MSN) marker. Mice were weighed daily to monitor health (data not shown).

### Progressively reduced forelimb motor performance in HD mice

**Figure 2.**
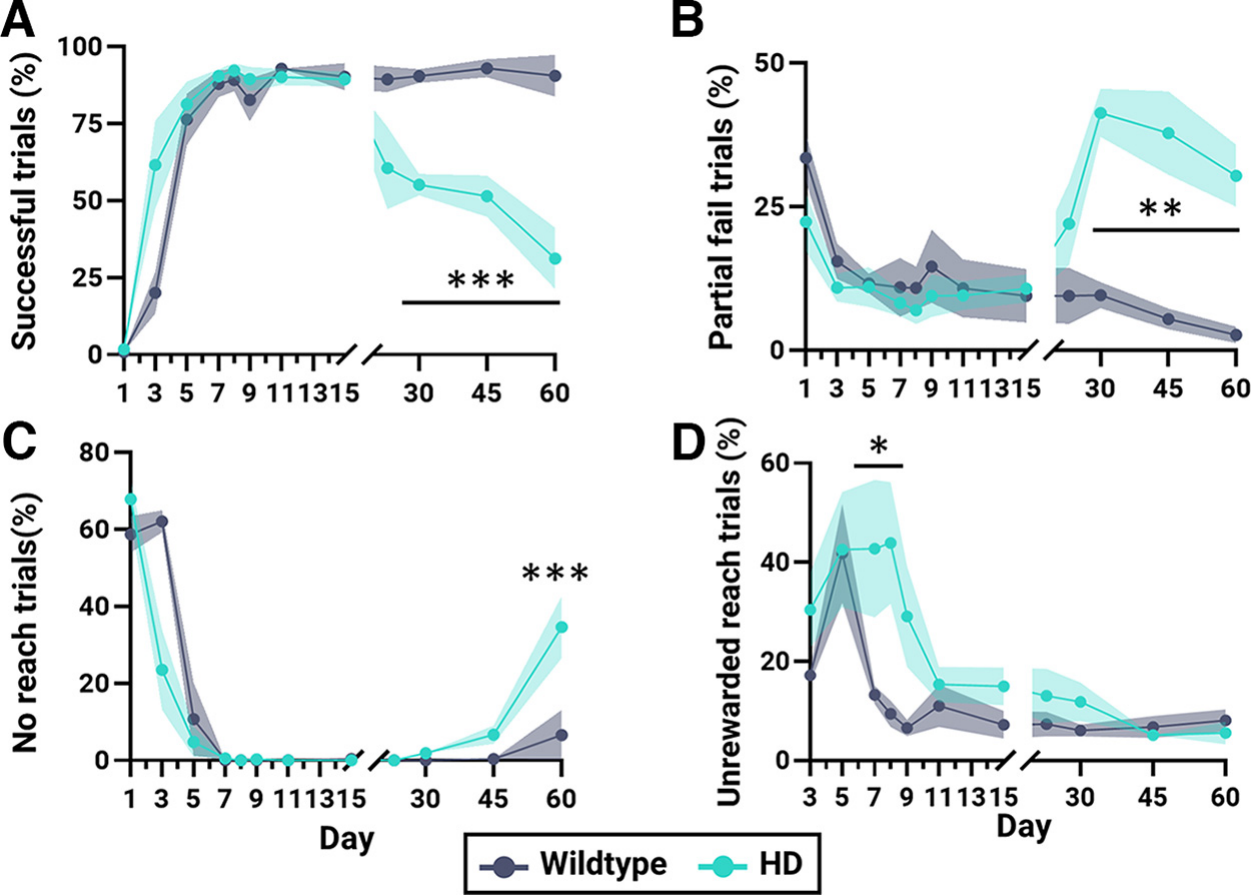
Water-reaching task behavioral categorization. HD (*n* = 6) and WT (*n* = 4) mice are denoted in teal and gray, respectively. ***A–C***, Percent of successful (***A***), partial fail (***B***), and no reach (***C***) trials to the total number of rewarded trials over time. ***D***, Percent of unrewarded reach trials (reaching occurs despite there being no reward) to the total number of nonrewarded trials over time. Shaded intervals denote SEM; ***p < 0.005, **p < 0.01, *p < 0.05. See Extended Data Figure 2-1 for additional data.

**Figure 3.**
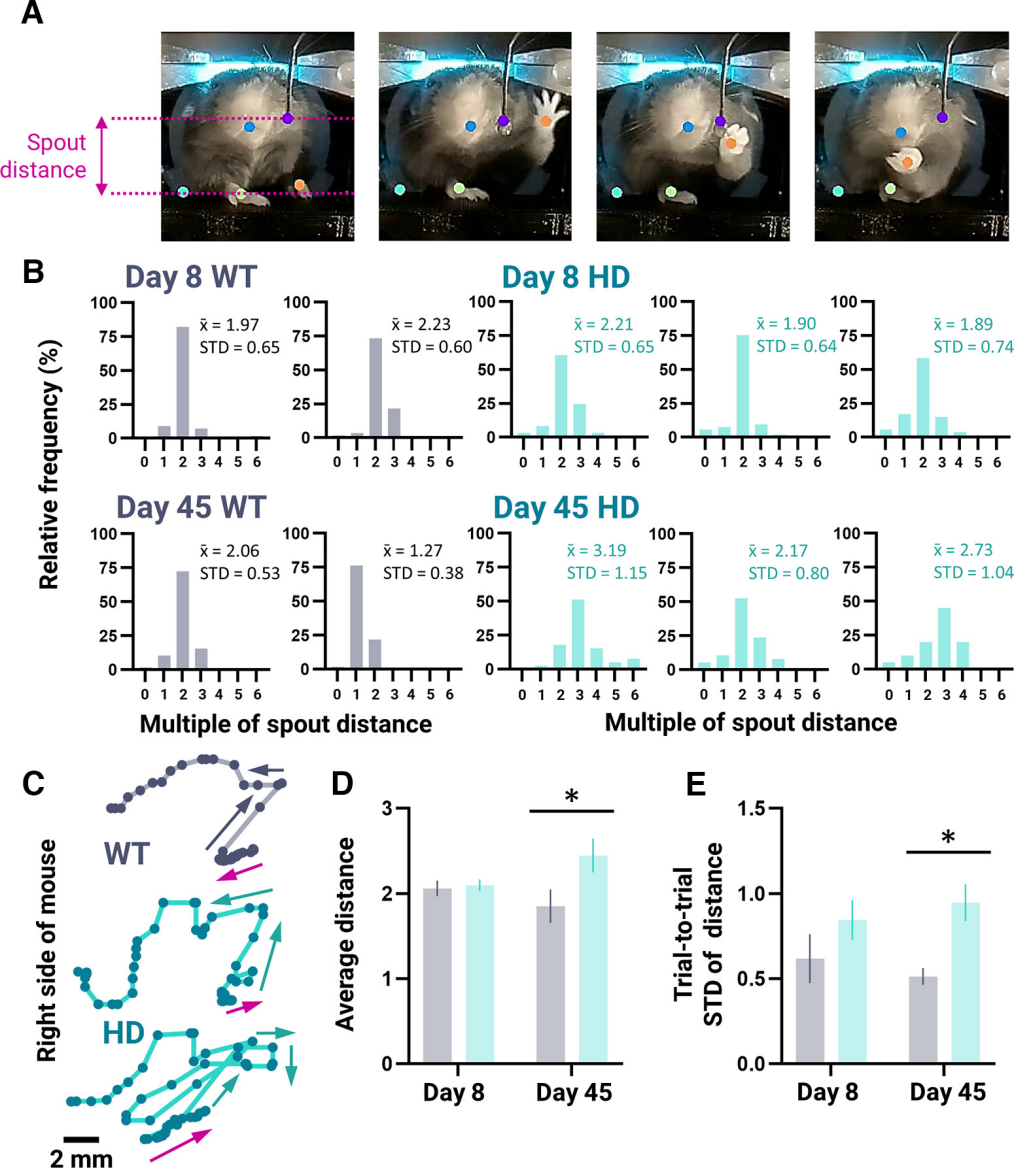
Kinematic analysis of successful trials. HD (*n* = 6) and WT (*n* = 4) mice are denoted in teal and gray, respectively. ***A***, Representative images depicting the mouse at rest and the reach-grasp-drink water-reaching movement. Dots represent either different body parts or equipment labeled for use in markerless pose estimation. The spout distance (calculated from the height of the platform to the height of the spout; for more details, see Materials and Methods) is depicted in purple. ***B***, Distribution of Euclidean distance traveled (water reward delivery to 1.1 s afterward) by the left paw during successful rewarded trials on day 8 (top graphs) and day 45 (bottom graphs) for representative WT and HD mice. The distance traveled in each trial was binned with intervals reflecting how many more times the path taken was compared with the spout distance (for more details, see Materials and Methods). Relative frequencies (%) of each bin are reported. Average Euclidean distance traveled ($\bar{\text{x}}$) and SD (STD) are indicated and reflect multiples of spout distance. ***C***, Representative left paw *X*, *Y* trajectories of WT (top trace) and HD (middle and bottom traces) mice performing a successful water-reaching movement during a rewarded trial on day 45. Arrows denote path of trajectory. Purple arrows indicate the start of the trajectory. ***D***, ***E***, Average Euclidean distance traveled across all successful trials (***D***) and trial-to-trial variability (SD; STD) of successful reaching trajectories (***E***) on days 8 and 45. Measurements are given in multiples of spout distance; ***D***, ***E*** are per mouse averages. Error bars denote SEM; *p < 0.05. See Extended Data Figure 3-1 for additional data.

Trial structure for the water-reaching task with alternating rewarded and nonrewarded trials is depicted in Figure 1*C*. Briefly, a visual cue was delivered 2 s after initiation of camera recording for all trials. For rewarded trials, an auditory cue and water drop was delivered 4 s later (6 s since start of the trial). For nonrewarded trials only an auditory cue was given with no water delivered. Water-reaching performance in both genotypes was quantified over 60 d (Fig. 2). Over time mice were trained during rewarded trials to reach forward toward the spout from their resting position (reach-to-grasp behavior), grasp the water drop then successfully bring the water to their mouth to drink (grasp-to-drink behavior; Fig. 3*A*; Movie 1, Movie 2). We refer to this overall as the “reach-grasp-drink” movement. Although no aversive punishment was given for reaching the spout during nonrewarded trials, no water reward was available on the spout making any attempts futile.

10.1523/ENEURO.0452-22.2022.f2-1 Extended Data Figure 2-1 Categorization of unsuccessful rewarded trials. Proportion of unsuccessful rewarded trial types (no reach: blue; groom: purple; partial fail: green; complete fail: dark teal) and total unsuccessful trials (line) to the total number of rewarded trials for HD (*n* = 6; ***A***) and WT (*n* = 4; ***B***) mice overtime. Error bars denote SEM. Grooming and complete fail trials in both genotypes were minimal with no statistical differences between genotypes. Download Figure 2-1, TIF file.

Movie 1. Representative WT mouse performing a successful trial. Simultaneous cortical widefield GCaMP imaging (ΔF/F) and water-reaching behavior video (front and side view) from a WT mouse performing a successful trial. Scale bar: 0.5 mm. 10.1523/ENEURO.0452-22.2022.video.1

Movie 2. Representative HD mouse performing a successful trial. Simultaneous cortical widefield GCaMP imaging (ΔF/F) and water-reaching behavior video (front and side view) from a HD mouse performing a successful trial. Scale bar: 0.5 mm. 10.1523/ENEURO.0452-22.2022.video.2

On day 1, there were minimal successful trials as both groups were learning the task (Fig. 2*A*). In both genotypes (day 1) the largest proportion of rewarded trials were spent not engaging with the task (no reach; WT: 58.7 ± 4.7%; HD: 67.9 ± 5.0; Fig. 2*C*) with the second largest proportion of trials spent reaching the water spout to swat the water away (partial fail; HD: 22.3 ± 5.1%; WT: 33.4 ± 4.2%; Fig. 2*B*). After performing successful trials for the first time on day 3 (20.1 ± 6.8%; Fig. 2*A*), WT mice engaged in frequent unrewarded reaching and/or groom-to-reach behavior on day 5 (41.8 ± 10.0%; Fig. 2*D*). WT mice however, quickly decreased unrewarded reaching behavior and by day 8 performed minimal unrewarded reach trials (9.4 ± 2.7%). WT mice achieved a near perfect success performance rate by day 8 (WT: 89.2 ± 3.7%; Fig. 2*A*).

Similar to WT mice, HD mice also after performing successful trials for the first time on day 3 (61.6 ± 14.3%; Fig. 2*A*) engaged in frequent unrewarded reaching and/or groom-to-reach behavior on day 5 (42.6 ± 11.6%; Fig. 2*D*). The frequency of unrewarded reaching however, persisted. Although HD mice also reached near perfect success performance rates for rewarded trials by day 8 (HD 92.3 ± 2.5%; Fig. 2*A*), significantly more unrewarded reaching was still present on this day compared with WT (WT: 9.4 ± 2.7%; HD: 43.9 ± 12.3%; *p* = 0.0169). By day 11, the unrewarded reach trials in HD mice decreased to the same frequency as seen in WT mice.

Over time WT mice were able to maintain their high success rate until at least day 60 (Fig. 2*A*). HD mice however, experienced a progressive decline in successful trials. By day 60, the successful performance rate was 31.1 ± 10.0% for HD mice. No significant changes in weight were seen throughout the entire behavioral testing timeline indicating water restriction was not the cause of HD mice performance decline (data not shown).

Unsuccessful trials were divided and scored as either no reach, groom, partial fail and complete fail trials (Extended Data Fig. 2-1). Partial fail scores denote trials where the mouse made contact with the spout and removed the water drop from the spout but was unable to retain and drink the water drop (successful reach-to-grasp performance but failed grasp-to-drink performance). Complete fail scores denote trials where the mouse lifted their paw in a reaching behavior but the paw did not make contact with the spout (failed reach-to grasp performance).

The low prevalence of complete fail trials for the duration of behavioral testing for HD mice (Extended Data Fig. 2-1*A*) suggest that failure to perform the reach-to-grasp segment of the forelimb movement does not explain the decline in successful performance. HD mice, however, develop a significant increase in partial fail trials compared with WT mice by day 30 (WT: 9.6 ± 2.2%; HD: 41.3 ± 4.2%; *p* = 0.0024; Fig. 2*B*), suggesting instead the grasp-to-drink segment of the movement was impaired. By day 60, HD mice also developed a significant increase in no reach trials compared with WT mice (WT: 6.5 ± 6.5%; HD: 34.6 ± 8.1%; *p* = 0.0007; Fig. 2*C*). Throughout the whole duration of behavioral testing, mice in both genotypes spent a minimal number of rewarded trials grooming (Extended Data Fig. 2-1). In all, behavior categorization revealed deficits in event sequence and forelimb function in HD mice.

### Increased distance and variable forelimb reaching movement in HD mice

On average, WT mice were able to obtain the water drop 1.0 ± 0.1 s after reward delivery. As such, markerless pose estimation was used to track the left paw from the time of water reward delivery to 1.1 s afterward (Fig. 3). Euclidean distance traveled by the left paw during successful trials is presented as multiples of the spout distance over this fixed period of time. Sample paired distribution of reaching trajectory distances for two WT (gray) and three HD (teal) mice on day 8 (top panels) and day 45 (bottom panels) with corresponding average Euclidean distance and trial-to-trial SD are shown (Fig. 3*B*; all mice are shown in Extended Data Fig. 3-1). Unlike on day 8, when the average Euclidean distance traveled by the left paw was the same in both genotypes (WT: 2.1 ± 0.1; HD: 2.1 ± 0.1 spout distances; *p* = 0.9811), the left paw of HD mice traveled a greater distance during the reach on day 45 than WT mice (WT: 1.9 ± 0.2; HD: 2.4 ± 0.2 spout distances; *p* = 0.0320; Fig. 3*D*). Sample left paw reaching trajectories on day 45 are shown for WT and HD mice (Fig. 3*C*). The variability in reaching distances for all successful trials on day 8 (measured as the SD) was not statistically different between genotypes (WT: 0.62 ± 0.14; HD: 0.84 ± 0.12 spout distances; *p* = 0.3397; Fig. 3*E*). On day 45, however, HD mice displayed a greater variability in reaching trajectory distances than WT mice (WT: 0.51 ± 0.05; HD: 0.95 ± 0.11; *p* = 0.0360).

10.1523/ENEURO.0452-22.2022.f3-1 Extended Data Figure 3-1 Euclidean distance distribution for successful trials on day 8 and day 45. Distribution of Euclidean distance traveled by the left paw (water reward delivery to 1.1 s afterwards) during successful rewarded trials on day 8 (top graphs) and day 45 (bottom graphs) for all WT (gray) and HD (teal) mice. The distance traveled in each trial was binned with intervals reflecting how many more times the path taken was compared to the spout distance (calculated from the height of the platform to the height of the spout; for more details, see Materials and Methods). Relative frequencies (%) of each bin are reported. Average Euclidean distance traveled (x̄) and SD (STD) are indicated in multiples of spout distance. Download Figure 3-1, TIF file.

### Changes in cortical activity dynamics during reaching over time in HD mice

**Figure 4.**
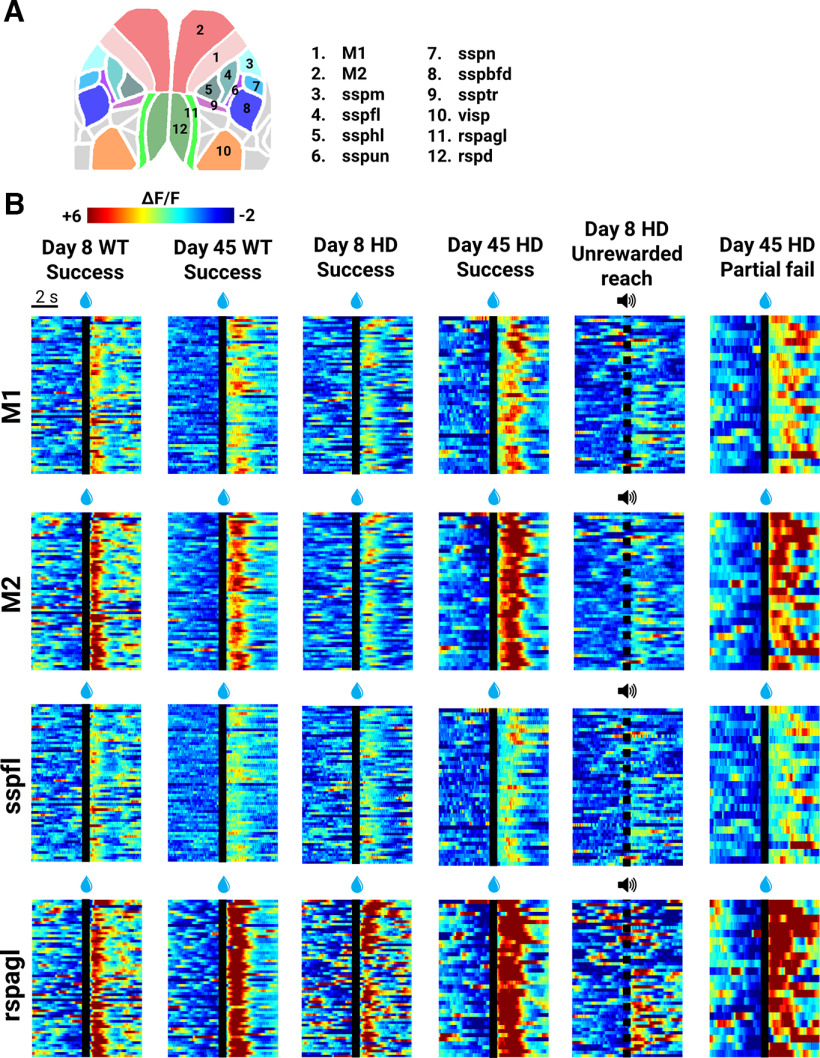
Representative trial-to-trial GCaMP cortical activity in regions of interest. ***A***, Cartoon depicts regions of interest investigated in subsequent analyses. ***B***, Representative trial-to-trial heat-map of GCaMP (ΔF/F) cortical activity in contralateral M1 (primary motor), M2 (secondary motor), sspfl (somatosensory forelimb), and rspagl (retrosplenial lateral agranular) from a WT and HD mouse on day 8 and day 45 for success, unrewarded reach, and/or partial fail trials. Individual trials are stacked in rows. Time of the water reward (for rewarded trials) and tone (for nonrewarded trials) is denoted with a black line. See Extended Data Figures 4-1, 4-2, and 4-3 for additional data.

The brain-to-atlas approach in MesoNet (Xiao et al., 2021) was used to register cortical images to a common atlas using predicted cortical landmarks. Regions of interest (ROIs) were then defined (Fig. 4*A*; ROIs are color and number labeled). Sample heat maps of trial-to-trial cortical ΔF/F for select ROIs during successful, unrewarded reach and/or partial fail trials on day 8 and/or 45 from a WT and HD mouse are shown (Fig. 4*B*; all mice are shown in Extended Data Figs. 4-1, 4-2, 4-3). Across genotypes, widespread activation of M1, M2, rspagl, and to a lesser degree sspfl was consistently apparent for all successful and partial fail trials after water drop presentation (Fig. 4*B* and Extended Data Figs. 4-1, 4-2, 4-3). In successful trials, activation increased by day 45 compared with day 8 for HD but not WT mice.

10.1523/ENEURO.0452-22.2022.f4-1 Extended Data Figure 4-1 WT trial-to-trial GCaMP heat-map for successful trials. Success trial-to-trial heat-map of GCaMP (ΔF/F) cortical activity in contralateral M1 (primary motor), M2 (secondary motor), sspfl (somatosensory forelimb), and rspagl (retrosplenial lateral agranular) for all WT mice on day 8 and day 45. Individual trials are stacked in rows. Time of the water reward is denoted with a black line. Download Figure 4-1, TIF file.

10.1523/ENEURO.0452-22.2022.f4-2 Extended Data Figure 4-2 HD trial-to-trial GCaMP heat-map for successful trials. Success trial-to-trial heat-map of GCaMP (ΔF/F) cortical activity in contralateral M1 (primary motor), M2 (secondary motor), sspfl (somatosensory forelimb), and rspagl (retrosplenial lateral agranular) for all HD mice on day 8 and day 45. Individual trials are stacked in rows. Time of the water reward is denoted with a black line. Download Figure 4-2, TIF file.

10.1523/ENEURO.0452-22.2022.f4-3 Extended Data Figure 4-3 HD trial-to-trial GCaMP heat-map for unrewarded reach and partial fail trials. Unrewarded reach and partial fail trial-to-trial heat-map of GCaMP (ΔF/F) cortical activity in contralateral M1 (primary motor), M2 (secondary motor), sspfl (somatosensory forelimb), and rspagl (retrosplenial lateral agranular) for all HD mice on day 8 and day 45, respectively. Individual trials are stacked in rows. Time of the water reward (for partial fail trials) and tone (for unrewarded reach trials) is denoted with a black line. Download Figure 4-3, TIF file.

**Figure 5.**
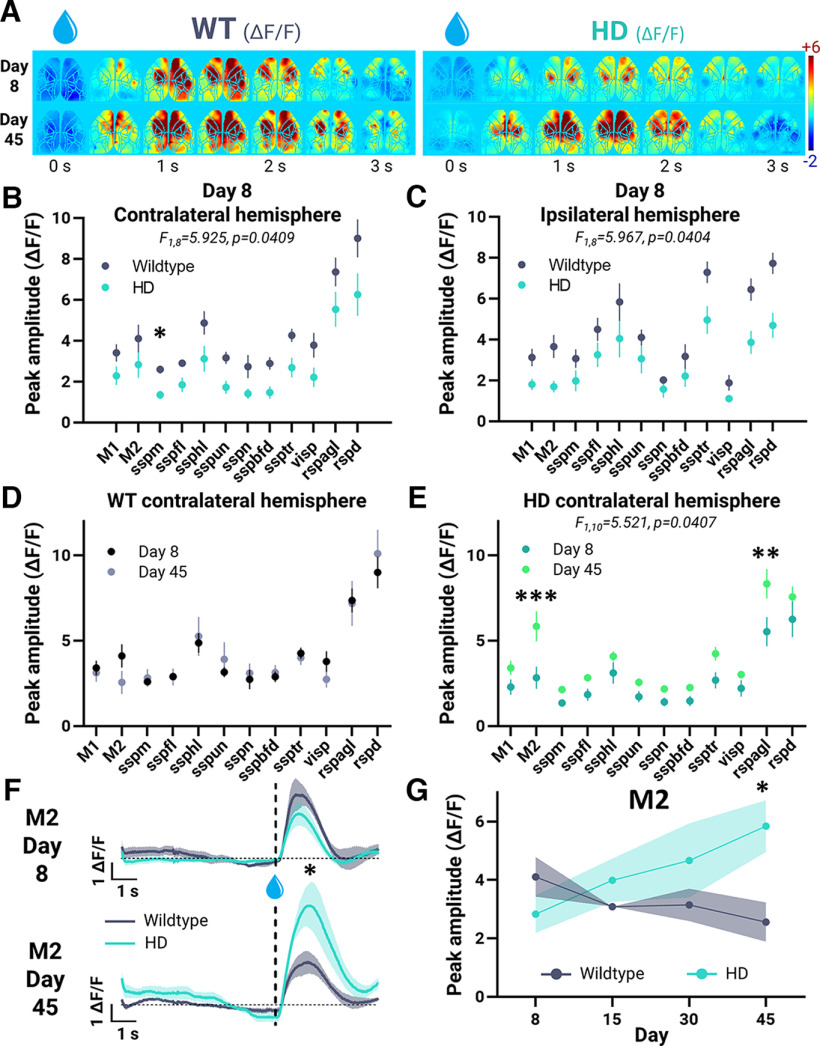
Longitudinal mesoscale GCaMP imaging of the cortex during water-reaching. HD (*n* = 6) and WT (*n* = 4) mice are denoted in teal and gray, respectively. 5 × 5 pixel regions are centered in regions of interest (ROIs) and examined. ***A***, Time series of cortical widefield GCaMP imaging (ΔF/F) from a representative WT (left panels) and HD (right panels) mouse on day 8 (top panels) and day 45 (bottom panels) during successful trials. ***B***, ***C***, Peak amplitude of regions of interest on day 8 for successful trials in the contralateral (*F*_(1,8)_ = 5.925, *p* = 0.0409, ANOVA; across genotype; ***B***) and ipsilateral (*F*_(1,8)_ = 5.967, *p* = 0.0404, ANOVA; across genotype; ***C***) hemisphere. ***D***, ***E***, Peak ΔF/F amplitude of ROIs in the contralateral hemisphere on day 8 and day 45 for WT (*F*_(1,72)_ = 0.0059, *p* = 0.9388, ANOVA; ***D***) and HD (*F*_(1,10)_ = 5.521, *p* = 0.0407, ANOVA; ***E***) mice. ***F***, Time course of M2 (secondary motor cortex) activation on day 8 (top panel) and day 45 (bottom panel). Vertical dotted line denotes time of water reward delivery. Horizontal dotted line denotes zero ΔF/F level. Significance reflects a difference in genotype peak response. ***G***, Corresponding change in peak ΔF/F amplitude of M2 over time. Error bars and shaded intervals denote SEM; ***p < 0.005, **p < 0.01, *p < 0.05. See Extended Data Figures 5-1, 5-2, and 5-3 for additional data.

Sample time series of GCaMP6 cortical widefield imaging on days 8 and 45 are shown in Figure 5*A* for a representative WT and HD mouse. On day 8, despite both HD and WT mice having comparable success rates (Fig. 2*A*) and reaching distances (Fig. 3*D*), genotype differences in cortical activity were apparent (Fig. 5*B*,*C*). Examining specific ROIs further revealed that the peak amplitude across all ROIs in both the contralateral and ipsilateral hemisphere was greater in WT compared with HD mice (contralateral: *F*_(1,8)_ = 5.925, *p* = 0.0409, ANOVA; ipsilateral: *F*_(1,8)_ = 5.967, *p* = 0.0404, ANOVA; Fig. 5*B*,*C*). Together, this indicates that on day 8, more extensive cortical activation associated with reaching was seen in WT compared with HD mice.

When examined longitudinally within genotypes (comparison of day 8 to day 45), the peak amplitude across all ROIs in the contralateral hemisphere increased in HD mice (*F*_(1,10)_ = 5.521, *p* = 0.0407, ANOVA; Fig. 5*E*) with no significant changes in WT mice (*F*_(1,72)_ = 0.006, *p* = 0.9388, ANOVA; Fig. 5*D*). In particular, the pixel regions centered in the contralateral secondary motor cortex (M2) and retrosplenial cortex lateral agranular part (rspagl) displayed significantly greater peak amplitude over time in HD mice (Fig. 5*E*). No significant changes in peak amplitude across all ROIs in the ipsilateral hemisphere were seen over time for WT (*F*_(1,6)_ = 0.026, *p* = 0.8770, ANOVA) and HD (*F*_(1,10)_ = 0.709, *p* = 0.4193, ANOVA) mice when comparing within genotypes (Extended Data Fig. 5-1).

10.1523/ENEURO.0452-22.2022.f5-1 Extended Data Figure 5-1 Ipsilateral mesoscale GCaMP imaging of the cortex during successful trials on days 8 and 45. Peak ΔF/F amplitude of ROIs in the ipsilateral hemisphere on day 8 and day 45 for WT (*F*_(1,6)_ = 0.0261, *p* = 0.8770, ANOVA; ***A***) and HD (*F*_(1,10)_ = 0.7094, *p* = 0.4193, ANOVA; ***B***) mice. Download Figure 5-1, TIF file.

When comparing genotypes (WT to HD) a significant difference in contralateral M2 peak activity was seen on day 45 (WT: 2.6 ± 0.7; HD: 5.8 ± 0.9; *p* = 0.0455; Fig. 5*G*). Figure 5*F* shows the average time course of contralateral M2 ΔF/F activation on days 8 and 45 for all mice. Genotype differences were also seen in contralateral sspm, sspbfd, and visp cortices on day 8 (Extended Data Fig. 5-2). Comparing WT to HD mice overtime further reveals differences in ipsilateral M2, rspagl and retrosplenial cortex, dorsal part (rspd; Extended Data Fig. 5-3). Overall, HD mice exhibited reduced peak cortical activity associated with successful reaching compared with WT mice on day 8. When compared over time, peak cortical activity increased in HD but not WT mice.

10.1523/ENEURO.0452-22.2022.f5-2 Extended Data Figure 5-2 Change in contralateral hemisphere ROI peak ΔF/F amplitude over time. Peak ΔF/F amplitude of ROIs in the contralateral hemisphere over time for WT (*n* = 4; gray) and HD (*n* = 6; teal) mice; **p < 0.01, *p < 0.05. Download Figure 5-2, TIF file.

10.1523/ENEURO.0452-22.2022.f5-3 Extended Data Figure 5-3 Change in ipsilateral hemisphere ROI peak ΔF/F amplitude over time. Peak ΔF/F amplitude of ROIs in the ipsilateral hemisphere over time for WT (*n* = 4; gray) and HD (*n* = 6; teal) mice; **p < 0.01, *p < 0.05. Download Figure 5-3, TIF file.

### Impulsive and failed trials performed by HD mice

**Figure 6.**
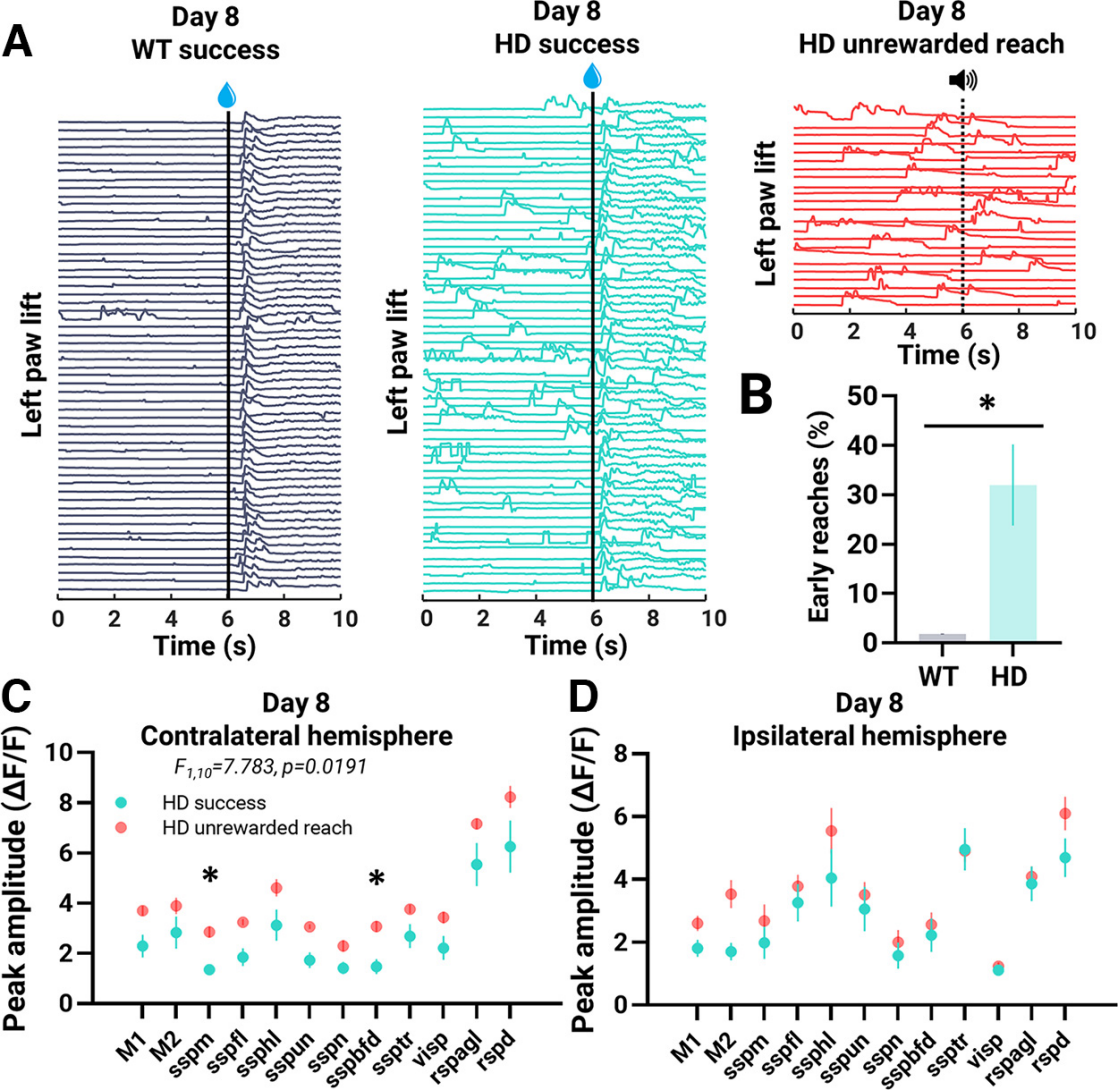
Impulsive reaches performed by HD mice on day 8. WT (*n* = 4) successful trials are denoted in gray. HD (*n* = 6) successful and unrewarded reach trials are denoted in teal and red, respectively. ***A***, Representative trial-to-trial traces of left paw lift (*y*-direction movement of the left paw) from a WT and HD mouse on day 8 for successful and/or unrewarded reach trials. Individual trials are stacked in rows. Time of the water reward (for rewarded trials) and tone (for nonrewarded trials) is denoted with a black line. ***B***, Percent of early reaches (reaches which occurred before water drop reward delivery) to the total number of successful trials. ***C***, ***D***, Peak amplitude of regions of interest in the contralateral (*F*_(1,10)_ = 7.783, *p* = 0.0191, ANOVA; ***C***) and ipsilateral (*F*_(1,10)_ = 1.531, *p* = 0.2442, ANOVA; ***D***) hemisphere for different HD trial types. Error bars denote SEM; *p < 0.05. See Extended Data Figure 6-1 for additional data.

Although both genotypes learned to perform successful trials by day 8 (Fig. 2*A*), HD mice also engaged in noncued impulsive reaching behavior during both rewarded and nonrewarded trials. Successful rewarded trials with early reaches, defined as reaches that occurred before presentation of the water drop, were more prevalent in HD than WT mice on day 8 (WT: 1.8 ± 0.1%; HD 32.0 ± 8.2%; *t* = 2.937; df = 8; *p* = 0.0188; % of total successful trials; Fig. 6*A*,*B*). Representative left paw lift (*y*-direction traces of left paw movement) in Figure 6*A* depicts trial-to-trial prevalence of early reaches in a WT (gray) and HD (teal) mouse. HD mice also continued to engage in reaching behavior during nonrewarded trials (Fig. 6*A*, red panel) for more days than WT mice (Fig. 2*D*).

10.1523/ENEURO.0452-22.2022.f6-1 Extended Data Figure 6-1 Mesoscale GCaMP imaging of the cortex during success and fail trials performed by HD mice on Day 45. HD (*n* = 6) success and fail trials are denoted in green and blue, respectively. ***A***, ***B***, Peak amplitude of regions of interest in the contralateral (*F*_(1,10)_ = 2.540, *p* = 0.1421, ANOVA; ***A***) and ipsilateral (*F*_(1,10)_ = 0.2573, *p* = 0.6230, ANOVA; ***B***) hemisphere for different trial types. ***C***, Area activated across the entire trial duration for successful and failed trials. The threshold was set at 4× SD (STD) of the baseline. No significance between trial types. Download Figure 6-1, TIF file.

Cortical activity underlying unrewarded reach trials was compared with successful reaches for HD mice on day 8. The peak amplitude of the GCaMP ROIs was first determined after the cue associated with a water reward for both trial types and was found to be reduced in unrewarded reach compared with successful trials (data not shown). Although successful trials sometimes included early reaches in HD mice, all these trials were characterized by a reach movement timed after the water drop (Fig. 6*A*, teal); however, reaches during nonrewarded trials occurred throughout the trial duration (Fig. 6*A*, red). Therefore, cortical activity underlying these unrewarded reaches was also examined across the entire trial duration and compared with successful reaches which occurred after the water reward (for more details, see Materials and Methods; Fig. 6*C*,*D*). In HD mice on day 8, the peak amplitude of all the ROIs in the contralateral (*F*_(1,10)_ = 7.783, *p* = 0.0191, ANOVA; Fig. 6*C*), but not the ipsilateral (*F*_(1,10)_ = 1.531, *p* = 0.2442, ANOVA; Fig. 6*D*) hemisphere was significantly increased in unrewarded reach trials compared with successful reaches. In particular, the peak cortical activity at the contralateral sspm and sspbfd cortices was significantly increased in unrewarded reach trials (Fig. 6*C*).

Overtime, unlike WT mice which maintained their successful performance at the water-reaching task, HD mice experienced a decline in performance (Fig. 2*A*). No differences in cortical GCaMP peak amplitude or area activated was seen when comparing successful to failed reach trials performed by HD mice on day 45 (Extended Data Fig. 6-1). Consistent reaching during nonrewarded trials ceased by day 11 in HD mice (Fig. 2*D*). In all, HD mice at ∼5.5 months have an event sequence defect as evident by impulsive reaches.

### Phenotyping of gross motor defects and HD pathology

**Figure 7.**
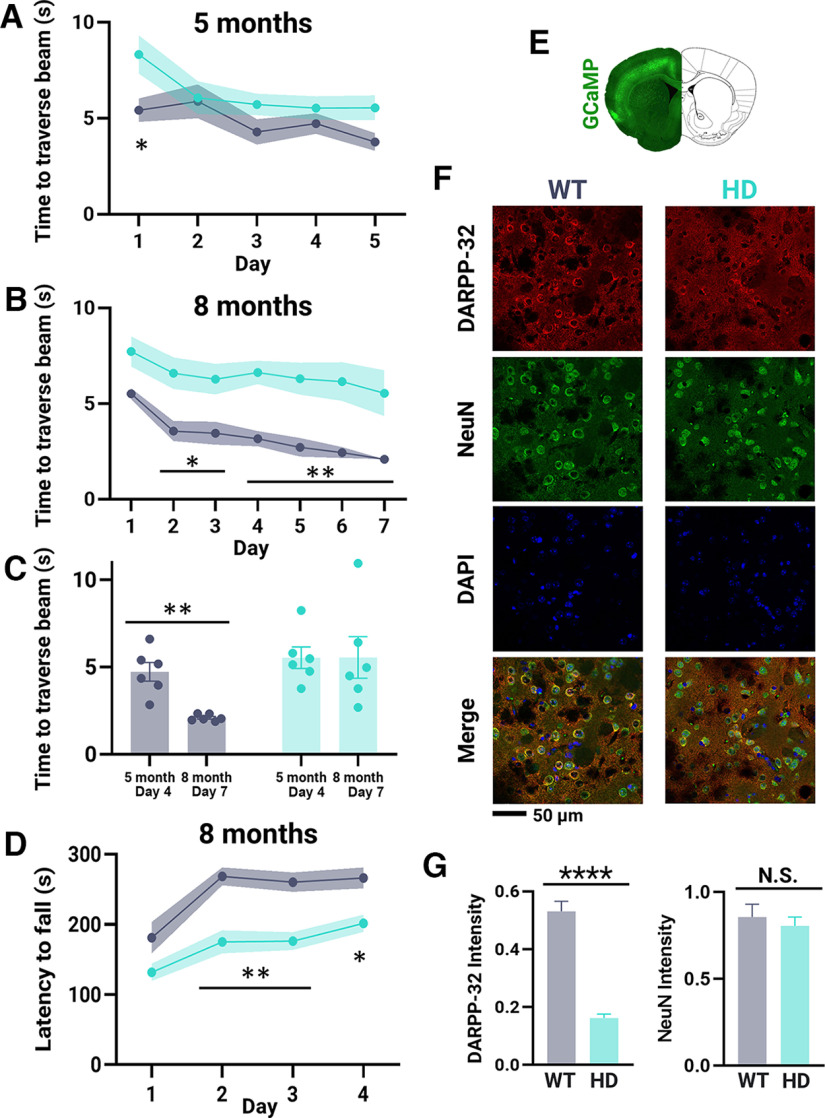
Tapered beam and rotarod gross motor assessment and postmortem immunohistochemistry staining. HD and WT mice are denoted as teal and gray, respectively. ***A***, ***B***, Time to traverse the tapered beam determined before (∼5 months; ***A***) and after (∼8 months; ***B***) water-reaching testing for HD (*n* = 6) and WT (*n* = 6) mice. ***C***, Time to traverse the tapered beam on the first day after completion of tapered beam learning (mice age: ∼5 month; day of testing: 4) compared with the last testing date (mice age: ∼8 month; day of testing: 7) for HD and WT mice. ***D***, Latency to fall from the rotarod determined after water-reaching testing (∼8 months) for HD (*n* = 6) and WT (*n* = 6) mice. ***E***, Representative Thy1-GCaMP6s coronal slice. DARPP-32 intensity was quantified in the striatum. ***F***, Representative images of DARPP-32, NeuN, and DAPI staining in the striatum with a merged overlay from a WT and HD mouse. ***G***, DARPP-32 and NeuN intensity in the striatum of HD (*n* = 4) compared with WT (*n* = 4) mice. Error bars and shaded intervals denote SEM; ****p < 0.0001, **p < 0.01, *p < 0.05; N.S., statistically nonsignificant, as determined by two-way ANOVA and Šídák’s multiple comparisons *post hoc* test for tapered beam and rotarod tests and unpaired *t* test for immunohistochemistry staining.

HD motor phenotype assessment using the water-reaching task was compared with gross motor tests (Fig. 7*A–D*). Before the water-reaching assessment, both genotypes with the exception of day 1, spent on average the same time traversing the tapered beam (Fig. 7*A*) indicating HD mice likely do not have a gross motor deficit at approximately five months of age. However, persistent unrewarded reach trials (Fig. 2*D*), early reaches during successful trials (Fig. 6*B*) and reduced peak cortical activity associated with successful trials (Fig. 5*B*,*C*) were revealed in the water-reaching assessment in HD but not WT mice at the same age.

All mice were also subjected to a second round of tapered beam testing after water-reaching assessment at approximately eight months of age (Fig. 7*B*). During this second testing phase, with the exception of day 1, WT mice traversed the beam significantly faster than HD mice. We then compared the performance of the mice during the first round of testing (mice aged approximately five months) with the second round (mice aged approximately eight months). Day 4 has previously been used as the first testing day after successful learning of the task (Ardesch et al., 2017). Comparison of day 4 during the first round of testing to the last assessment day (day 7) during the second round of testing, revealed that WT mice traversed the beam in a faster time by the last assessment day (first testing round day 4: 4.728 ± 0.532 s; second testing round day 7: 2.097 ± 0.077 s; *p* = 0.0044; Fig. 7*C*). For HD mice, the time to traverse the beam did not change between the first (day 4: 5.541 ± 0.614 s) and second (day 7: 5.550 ± 1.192 s) round of testing (Fig. 7*C*). After water-reaching training, HD mice also displayed impaired performance on the accelerating rotarod task as determined by a decreased latency to fall compared with WT (Fig. 7*D*). Together, the second round of tapered beam and rotarod testing suggest HD mice have reached the gross motor manifest stage of disease. This is consistent with the decreased success rate seen by the end of the water-reaching task in HD mice (Fig. 2*A*).

Finally, striatal MSNs which make up 95% of all neurons in the striatum were immunostained for DARPP-32 (Fig. 7*E–G*). Consistent with previous literature (Peng et al., 2016; Southwell et al., 2016), a significant decrease in DARPP-32 (relative intensity WT: 0.531 ± 0.035; HD: 0.162 ± 0.014; *t* = 9.765; df = 6; *p* < 0.0001) but not NeuN (relative intensity WT: 0.86 ± 0.07; HD: 0.81 ± 0.05; *t* = 0.5644; df = 6; *p* = 0.5930) intensity was seen in HD compared with WT mice (Fig. 7*G*). Overall, at approximately five months, early HD phenotype was apparent in the water-reaching task but not the tapered beam test. Gross motor HD deficits determined using rotarod and tapered beam tests, as well as HD pathology revealed through reduced striatal DARPP-32 expression were consistent with forelimb reaching deficits at approximately eight months.

## Discussion

**Figure 8.**
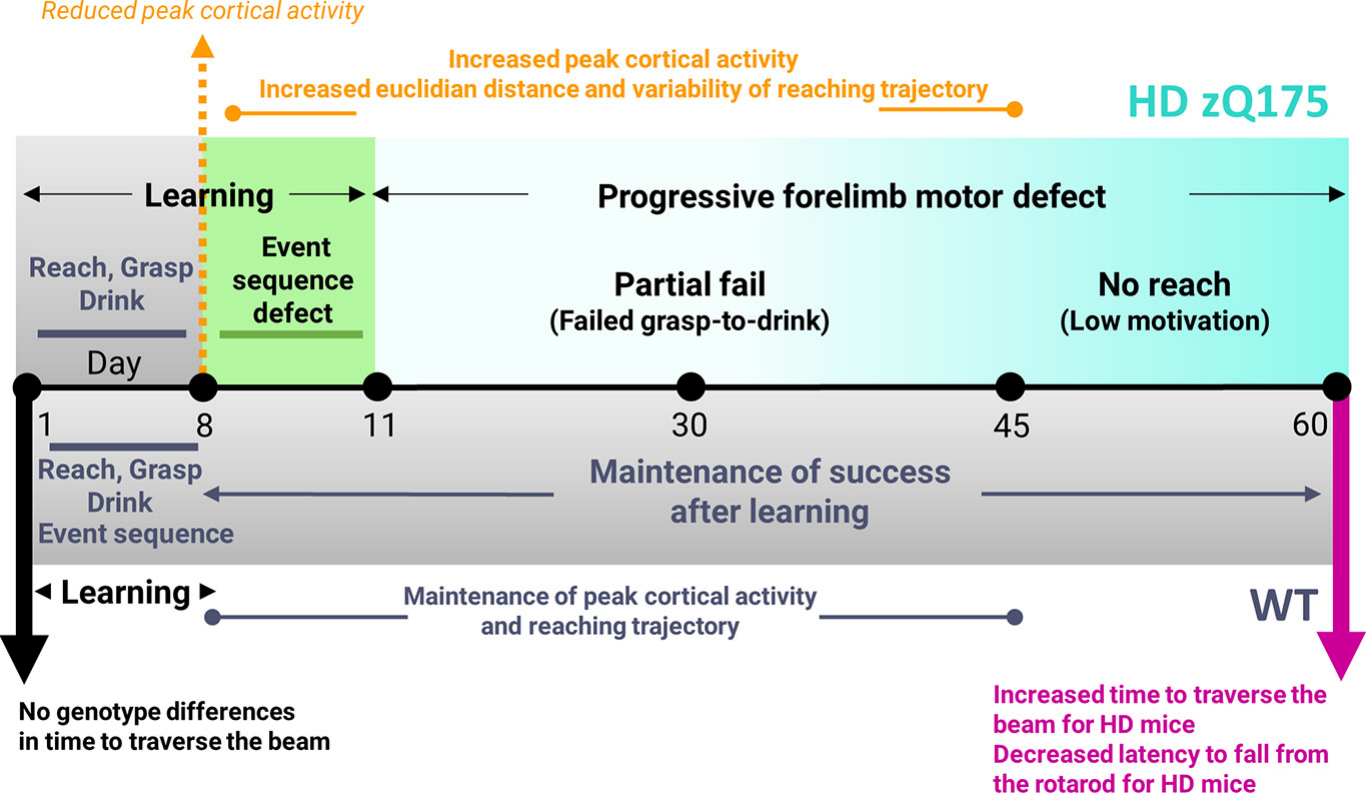
Schematic summary of altered cortical activity and motor defects in HD mice. Timeline of HD (top) and WT (bottom) learning and performance in the water-reaching task. Gross motor tapered beam and rotarod tasks are included. WT mice learn both the reach-grasp-drink movement and task event sequence (alternating reward then nonrewarded trial) by day 8 (gray). Although HD mice also learn the reach-grasp-drink movement by day 8 (gray) HD mice show reduced cortical activation compared with WT mice. HD mice also have an event sequence defect as evident by impulsive reaches (green). Over time the peak cortical activity, Euclidean distance and variability of the reaching trajectory increases in HD mice but little to no change was seen in WT mice. Unlike WT mice, HD mice also do not maintain their rate of successful performance overtime. HD mice experience first a progressive increase in partial fail trials then an increase in no reach trials reflecting failed grasp-to-drink then low task engagement, respectively. Overall, this indicates a progressively worsening forelimb motor coordination defect (light to dark teal) in HD mice that was captured daily.

The shared evolutionary origin and characteristics of skilled forelimb movements between humans and rodents (Whishaw et al., 1992; Galiñanes et al., 2018) enable translational parallels to be drawn from preclinical mouse studies. In HD patients and presymptomatic carriers, deficits in motor learning, temporal sequencing and coordination of voluntary movements have been reported (Feigin et al., 2006; Klein et al., 2011; Shabbott et al., 2013). Using a water-reaching task, we reveal the presence of event sequence defects and progressive increases in cortical activity underlying forelimb deficits in the zQ175 HD mouse model (Fig. 8 for a summary of the results).

### Task acquisition and performance across genotypes

For most motor tasks, initial learning is accompanied by trial-to-trial variability, enabling spatial exploration and progress toward efficient task execution (Dhawale et al., 2019). Variability is subsequently reduced after strategy formation (Churchland et al., 2006). We observe similar features since by day 8, both HD and WT mice were able to successfully learn the reach-to-grasp movement. Although the movement was successfully learned by both genotypes, cortical activity underlying successful reaches was reduced in HD mice compared with WT. HD mice also required more days to learn the alternating reward/nonreward event sequence as evident by initial impulsive reaching. We speculate that the extended continuation of reaching behavior during nonrewarded trials until day 11 and the occurrence of early reaches on day 8, both in HD mice, could be explained by underlying cognitive defects that slow learning because of an inability to remember when to reach or failure to suppress motor movement. Disconnection of the cortex and striatum as well as overactivation of the striatal direct pathway have previously been reported in early symptomatic HD mice (Barry et al., 2022) and may contribute to the hyperactivity and this event sequence defect.

Over time HD mice experienced a significant drop in successful reaches compared with WT. The increased trial-to-trial variability seen in HD mice compared with WT mice on day 45 suggests that HD mice are attempting compensatory changes in reaching strategy at a time when they experienced a drop in performance. Consistent with this, positional error correction of the forelimb has previously been observed in consecutive reach trials (Becker et al., 2020). The significant increase in partial fail trials but not complete fail trials further suggests HD mice fail to engage in proper end-point fine motor corrections during the grasp-to-drink segment of the task (Elliott et al., 2001). Semi-flexed or closed paws have been shown to result in failed target reaching trials (Whishaw et al., 2018b) and could explain the decline in successful performance rates seen in HD mice. By day 60, decreased task engagement was seen indicating that movement defects in HD mice increased in severity and alternative reaching strategies were no longer sufficient to mediate continued motivation and task engagement.

### Bilateral engagement of mesoscale cortical circuits during reaching

Consistent with other studies that report global activation of the cortex and involvement of the ipsilateral hemisphere during limb movement (Heming et al., 2019; Soma et al., 2019; Brunner et al., 2020; Quarta et al., 2022), our results also revealed widespread cortical activation across both hemispheres during water-reaching. Although we did not see widespread enhanced cortical activity in HD mice as some work indicates (Arnoux et al., 2018; Burgold et al., 2019; Sepers et al., 2021) compared with WT (except in M2), global cortical activation associated with reaching increased over time in HD mice (but not WT). The lack of increased cortical activity may be because of differences in reaching task-performing awake versus anesthetized animals, HD mouse models and/or cortical areas examined. We speculate that this increase in cortical activity seen over time in HD mice may be driven by increases in average Euclidean distance of the reaching trajectory. The increased Euclidean distance seen in HD mice was a result of multiple subreach attempts that eventually led to successful task execution. Another explanation could involve changes in local inhibitory inputs (Cummings et al., 2009), spontaneous firing rates and/or activity of the striatum (Donzis et al., 2020) over time during water-reaching assessment.

Examining cortical regions of interest revealed genotypic differences in peak GCaMP6 cortical responses in M2, sspm, sspbfd, and visp of the contralateral hemisphere and rspagl, rspd and M2 of the ipsilateral hemisphere. Differences have been reported in tongue protrusions during freely moving pellet reaching between individual mice and trial types (Whishaw et al., 2018a). Although tongue protrusions were not evident in either WT or HD mice, adjustments to the tongue within the mouth, the mouth itself or whisking could explain the differences seen in sspm and sspbfd. Chemosensory, but not spatial or visual cues have been shown to guide water-reaching behavior (Galiñanes et al., 2018). The significance of visual area GCaMP activity differences observed across genotypes seen in this study necessitates further investigation into other cortical areas not typically examined in the context of forelimb reaching and other motor tasks. The retrosplenial cortex also showed genotype-specific changes and has been linked to spatial memory and navigation (Czajkowski et al., 2014; Milczarek et al., 2018). Retrosplenial cortices may be involved in learning and maintaining correct spatial orientation of the paw toward the target (water reward). Further work is needed to fully understand the role retrosplenial cortices play in forelimb reaching and its contributions to the HD phenotype.

Optogenetic cortical silencing has revealed the motor cortex is critical for the adjustment of complex grasping movements (Mohammed et al., 2020). Specifically, M2 has also been reported to encode movement distance and smoothness (Quarta et al., 2022). Our findings that HD mice fail to perform the grasp-to-drink portion of the movement (increased partial fail trials compared with WT) and have an increased average Euclidean distance in their reaching trajectory compared with WT likely explains the genotypic hyperactivity seen in HD contralateral M2 compared with WT and is consistent with the previously reported roles M2 plays in forelimb reaching. This M2 hyperactivity evident at the motor manifest, but not premanifest stage is analogous to increased striatal activity seen with phenotype progression in HD (YAC128) mice compared with age-matched WT mice during rotarod performance (Koch et al., 2022).

We acknowledge that epifluorescence widefield calcium imaging which we use to assess excitability has reduced temporal resolution compared with voltage sensitive dyes and is sensitive to artifacts associated with light scattering, hemodynamics and movement. ROIs generated using cortical image and landmark registration to a common atlas (Xiao et al., 2021) may also not represent the same regions as those determined functionally. To mitigate some of these limitations, strobing of green reflectance light was used to correct hemodynamic artifacts (Wekselblatt et al., 2016; Vanni et al., 2017; Xiao et al., 2017). The head-fixed set-up further reduced movement. Despite some limitations, our study has demonstrated the water-reaching task can reliably characterize forelimb motor defects and reveal aberrant cortical activity in HD mice.

Consistent with other studies examining HD gross motor defects (Southwell et al., 2016; Liu et al., 2021), we report no significant differences in tapered beam traverse time in approximately five-month aged zQ175 HD compared with WT mice. Although HD and WT mice achieved similar success rates at the water-reaching task at this age, event sequence defects and reduced peak cortical activity associated with successful trials were apparent suggesting the increased sensitivity of the water-reaching task to detect early HD motor phenotype at ∼5.5 months of age. Later, when HD mice experienced reduced performance at the water-reaching task, we and others also reported increased time to traverse the tapered beam, decreased latency to fall from the rotarod, and decreased DARPP-32 expression in striatal MSNs (Smith, 2014; Peng et al., 2016; Southwell et al., 2016; Liu et al., 2021).

Future studies could examine the contribution of diverse cortical areas (such as retrosplenial, visual, and somatosensory) and subcortical regions [such as the striatum (Brunner et al., 2020), cerebellum (Guo et al., 2021) and thalamus (Sauerbrei et al., 2020)] to forelimb tasks in mouse models of HD and other movement disorders. Therapeutic rescue of the HD phenotype using optogenetics and parsing the contribution of direct-indirect striatal (Reiner et al., 1988; Albin et al., 1992; Barry et al., 2018) and M2 cortico-striatal pathways (Fernández-García et al., 2020) would also enable mechanistic understanding of HD forelimb defects. The knock-in mice containing HD expanded triplet repeats used in our study would provide an ideal vehicle for these future experiments involving circuit-based manipulation by targeted expression of optogenetic agents. Through crossing with existing transgenic channelrhodopsin or halorhodopsin lines, or through the introduction of red-shifted opsins by AAVs, the role of specific interneuron classes could be determined and therapeutically modulated during water-reaching assessment. Overall, the ability of the water-reaching task to characterize HD phenotype especially at early stages of disease suggests it could be used to inform the onset of other movement disorders, therapeutic intervention windows and test drug efficacy.

## Synthesis

Reviewing Editor: Sunghee Cho, Burke Neurological Institute/Feil Family Brain and Mind Research Institute, Weill Cornell Medicine Decisions are customarily a result of the Reviewing Editor and the peer reviewers coming together and discussing their recommendations until a consensus is reached. When revisions are invited, a fact-based synthesis statement explaining their decision and outlining what is needed to prepare a revision will be listed below. The following reviewer(s) agreed to reveal their identity: NONE. Note: If this manuscript was transferred from JNeurosci and a decision was made to accept the manuscript without peer review, a brief statement to this effect will instead be what is listed below.

Synthesis

The study developed innovative behavior and imaging technique to assess neural activity and motor behavior in an experimental HD model. Both reviewers who have expertise in the field highlighted several aspects of study including innovative technique to gauge pathology, rigor of the study, and translational values. It was also noted on careful and through responses to the reviewers’ comments, which strengthen the manuscript. Detailed and specific comments from the reviewers are appended.

Reviewer 1

The authors have significantly revised the manuscript in response to the last round of review. Particularly, they have presented more clearly in the main manuscript that their new motor tasks and imaging methods can discover early motor deficits and associated neural activity changes that are not detected by traditional gross motor tasks. These revisions have significantly improved the paper.

Reviewer 2

In the revised version of the manuscript by Wang et al., the authors have addressed the comments thoroughly. The text and figures have improved in clarity, and the paper reads very well. The new behavior data in Fig. 6 is also helpful to illustrate the advantages of the authors’ approach. I understand that additional histological and molecular analyses are beyond the scope of the study.
