# Supplementary figures and images for: Water-Reaching Platform for Longitudinal Assessment of Cortical Activity and Fine Motor Coordination Defects in a Huntington Disease Mouse Model
Source: eNeuro. 2023 Jan 6;10(1):ENEURO.0452-22.2022. doi: 10.1523/ENEURO.0452-22.2022 (PMC9833054; doi:10.1523/ENEURO.0452-22.2022)

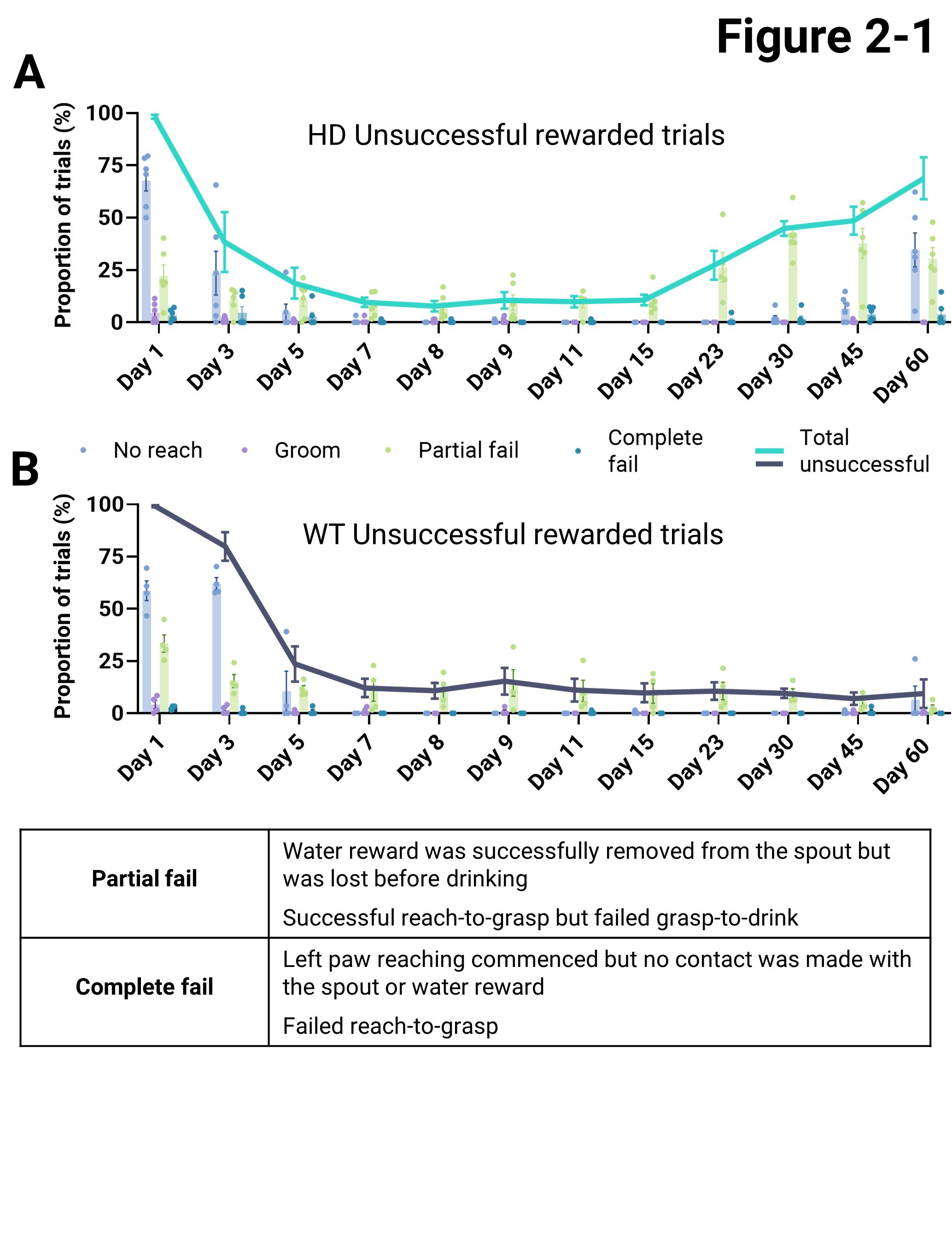

Supplement: Extended Data Figure 2-1 — Categorization of unsuccessful rewarded trials. Proportion of unsuccessful rewarded trial types (no reach: blue; groom: purple; partial fail: green; complete fail: dark teal) and total unsuccessful trials (line) to the total number of rewarded trials for HD (n = 6; A) and WT (n = 4; B) mice overtime. Error bars denote SEM. Grooming and complete fail trials in both genotypes were minimal with no statistical differences between genotypes. Download Figure 2-1, TIF file. [file enu-eN-MNT-0452-22-s01.tif]

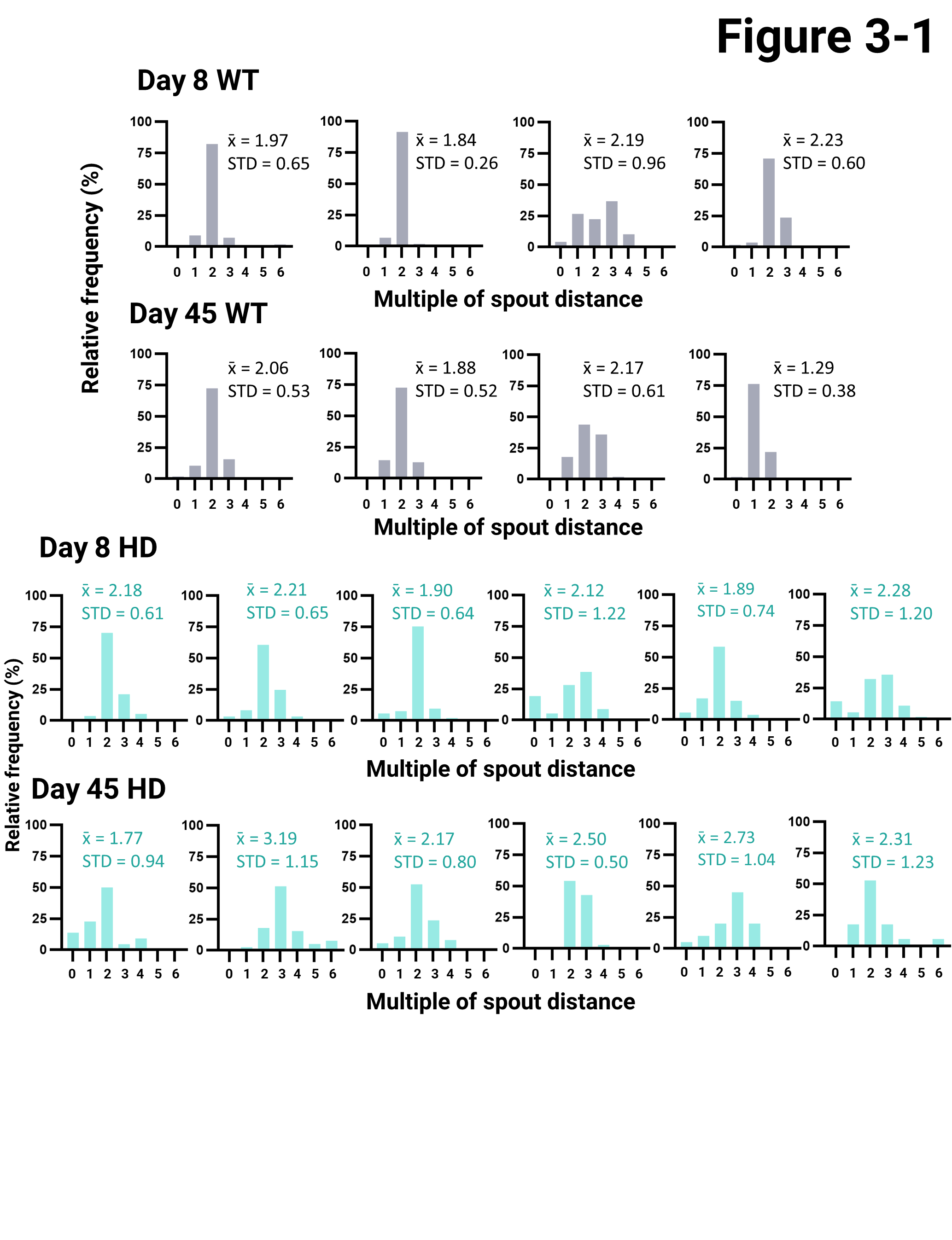

Supplement: Extended Data Figure 3-1 — Euclidean distance distribution for successful trials on day 8 and day 45. Distribution of Euclidean distance traveled by the left paw (water reward delivery to 1.1 s afterwards) during successful rewarded trials on day 8 (top graphs) and day 45 (bottom graphs) for all WT (gray) and HD (teal) mice. The distance traveled in each trial was binned with intervals reflecting how many more times the path taken was compared to the spout distance (calculated from the height of the platform to the height of the spout; for more details, see Materials and Methods). Relative frequencies (%) of each bin are reported. Average Euclidean distance traveled (x̄) and SD (STD) are indicated in multiples of spout distance. Download Figure 3-1, TIF file. [file enu-eN-MNT-0452-22-s02.tif]

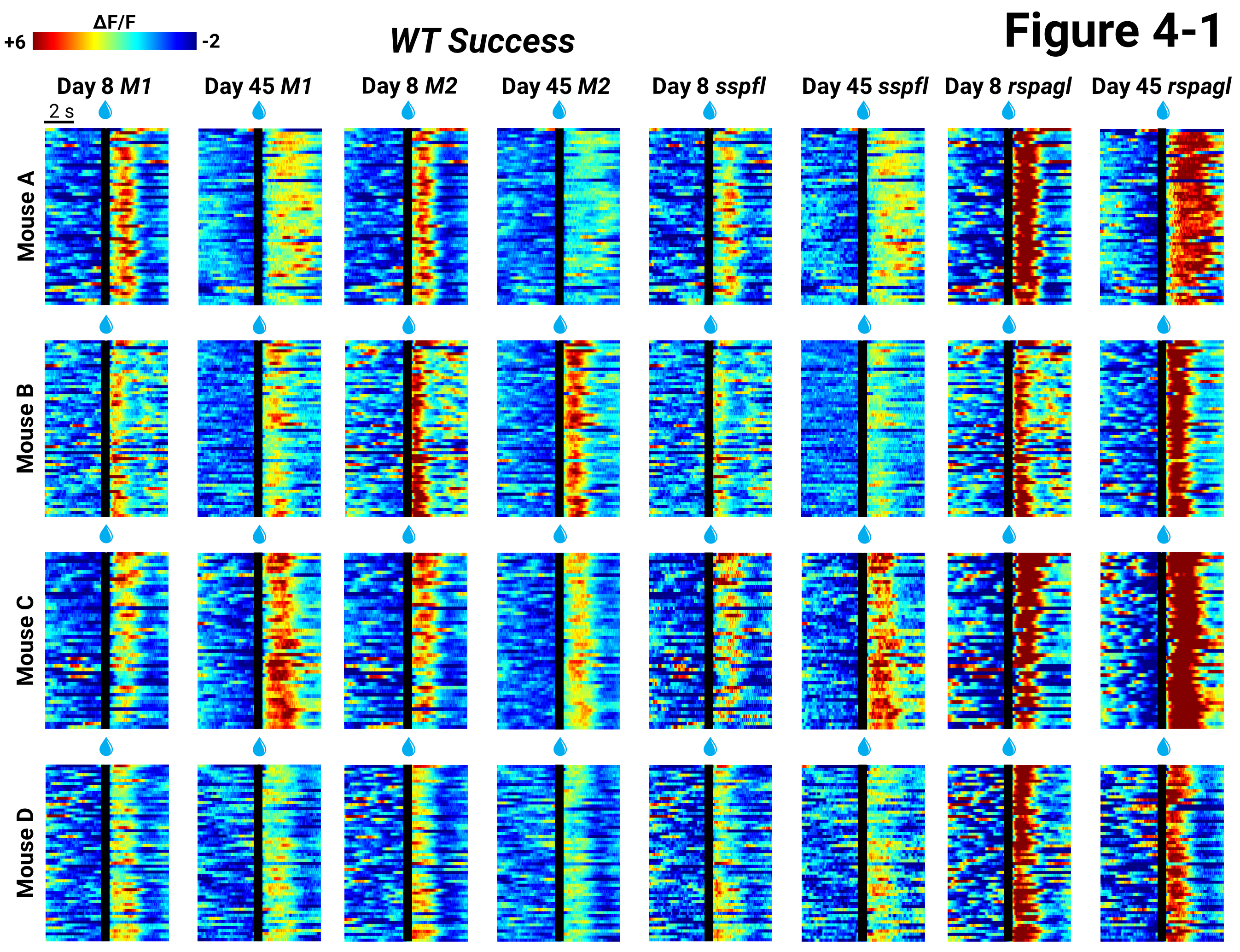

Supplement: Extended Data Figure 4-1 — WT trial-to-trial GCaMP heat-map for successful trials. Success trial-to-trial heat-map of GCaMP (ΔF/F) cortical activity in contralateral M1 (primary motor), M2 (secondary motor), sspfl (somatosensory forelimb), and rspagl (retrosplenial lateral agranular) for all WT mice on day 8 and day 45. Individual trials are stacked in rows. Time of the water reward is denoted with a black line. Download Figure 4-1, TIF file. [file enu-eN-MNT-0452-22-s03.tif]

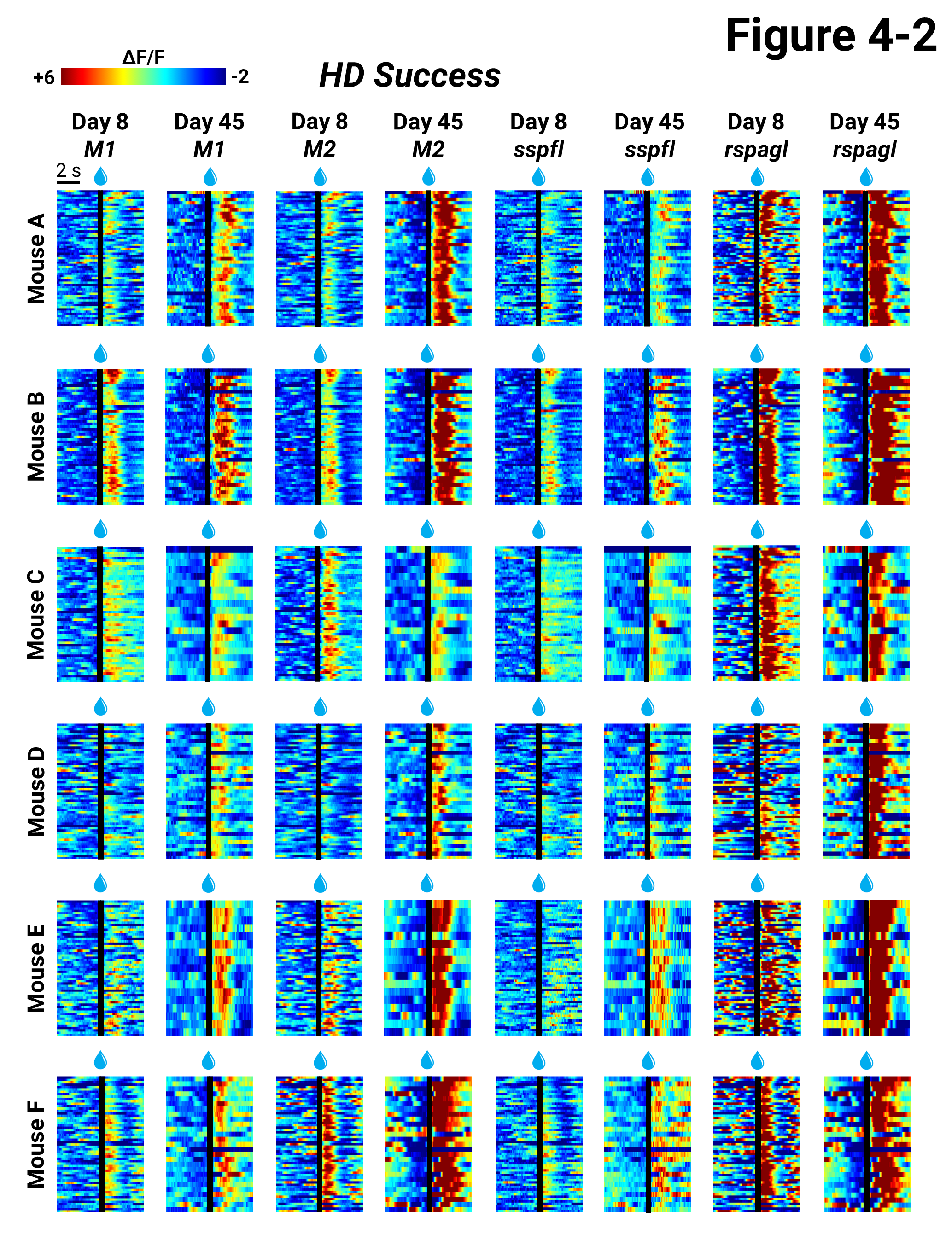

Supplement: Extended Data Figure 4-2 — HD trial-to-trial GCaMP heat-map for successful trials. Success trial-to-trial heat-map of GCaMP (ΔF/F) cortical activity in contralateral M1 (primary motor), M2 (secondary motor), sspfl (somatosensory forelimb), and rspagl (retrosplenial lateral agranular) for all HD mice on day 8 and day 45. Individual trials are stacked in rows. Time of the water reward is denoted with a black line. Download Figure 4-2, TIF file. [file enu-eN-MNT-0452-22-s04.tif]

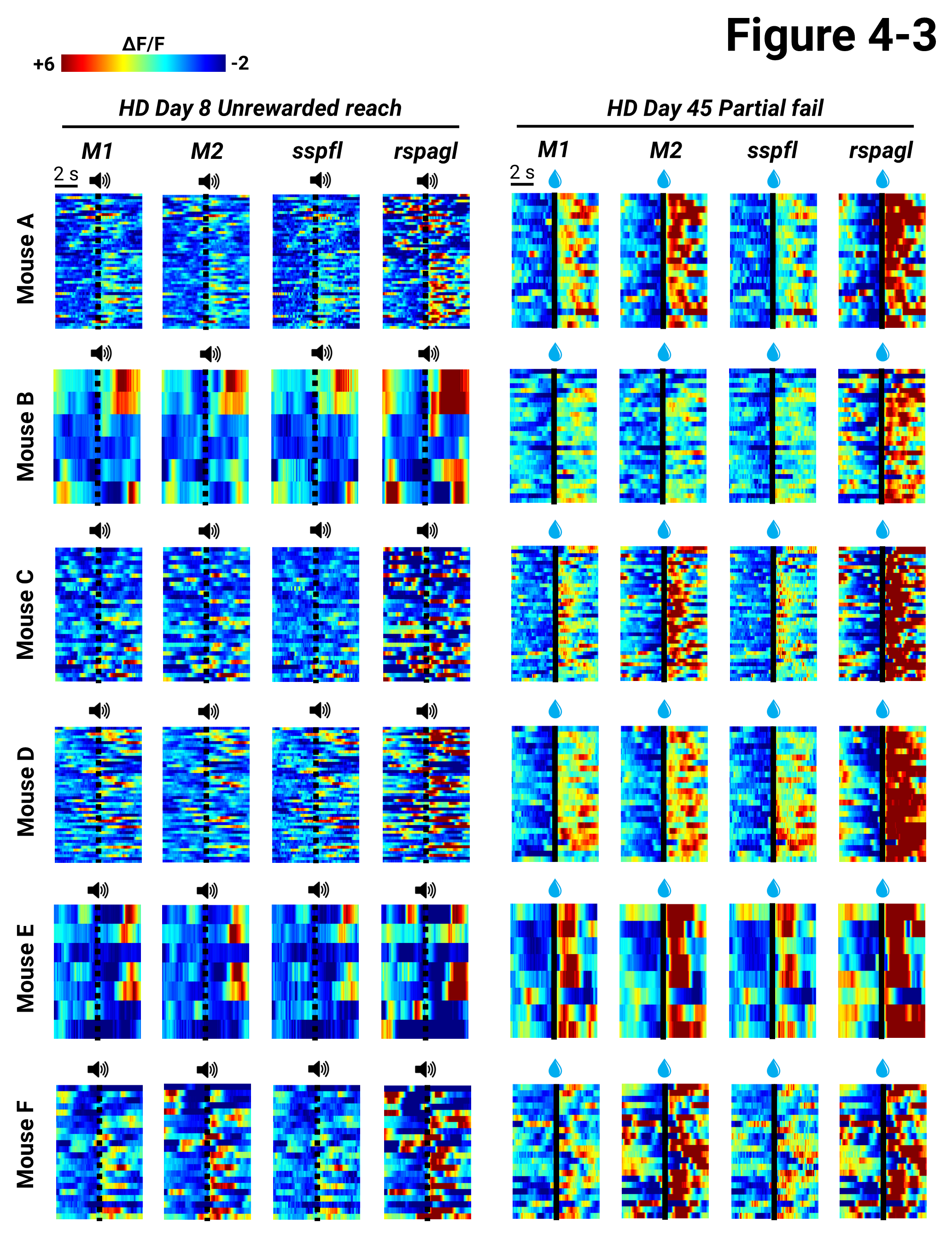

Supplement: Extended Data Figure 4-3 — HD trial-to-trial GCaMP heat-map for unrewarded reach and partial fail trials. Unrewarded reach and partial fail trial-to-trial heat-map of GCaMP (ΔF/F) cortical activity in contralateral M1 (primary motor), M2 (secondary motor), sspfl (somatosensory forelimb), and rspagl (retrosplenial lateral agranular) for all HD mice on day 8 and day 45, respectively. Individual trials are stacked in rows. Time of the water reward (for partial fail trials) and tone (for unrewarded reach trials) is denoted with a black line. Download Figure 4-3, TIF file. [file enu-eN-MNT-0452-22-s05.tif]

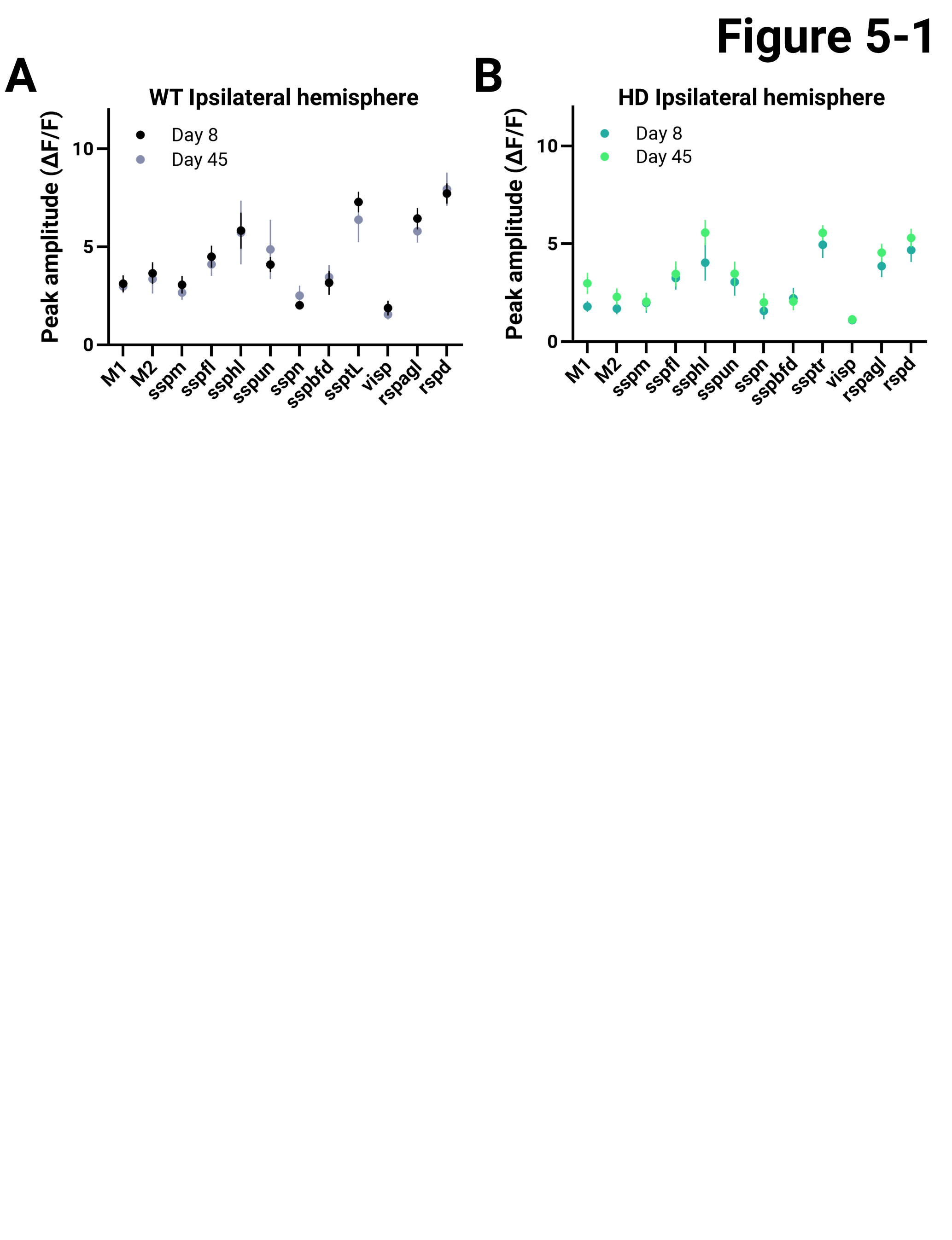

Supplement: Extended Data Figure 5-1 — Ipsilateral mesoscale GCaMP imaging of the cortex during successful trials on days 8 and 45. Peak ΔF/F amplitude of ROIs in the ipsilateral hemisphere on day 8 and day 45 for WT (F(1,6) = 0.0261, p = 0.8770, ANOVA; A) and HD (F(1,10) = 0.7094, p = 0.4193, ANOVA; B) mice. Download Figure 5-1, TIF file. [file enu-eN-MNT-0452-22-s06.tif]

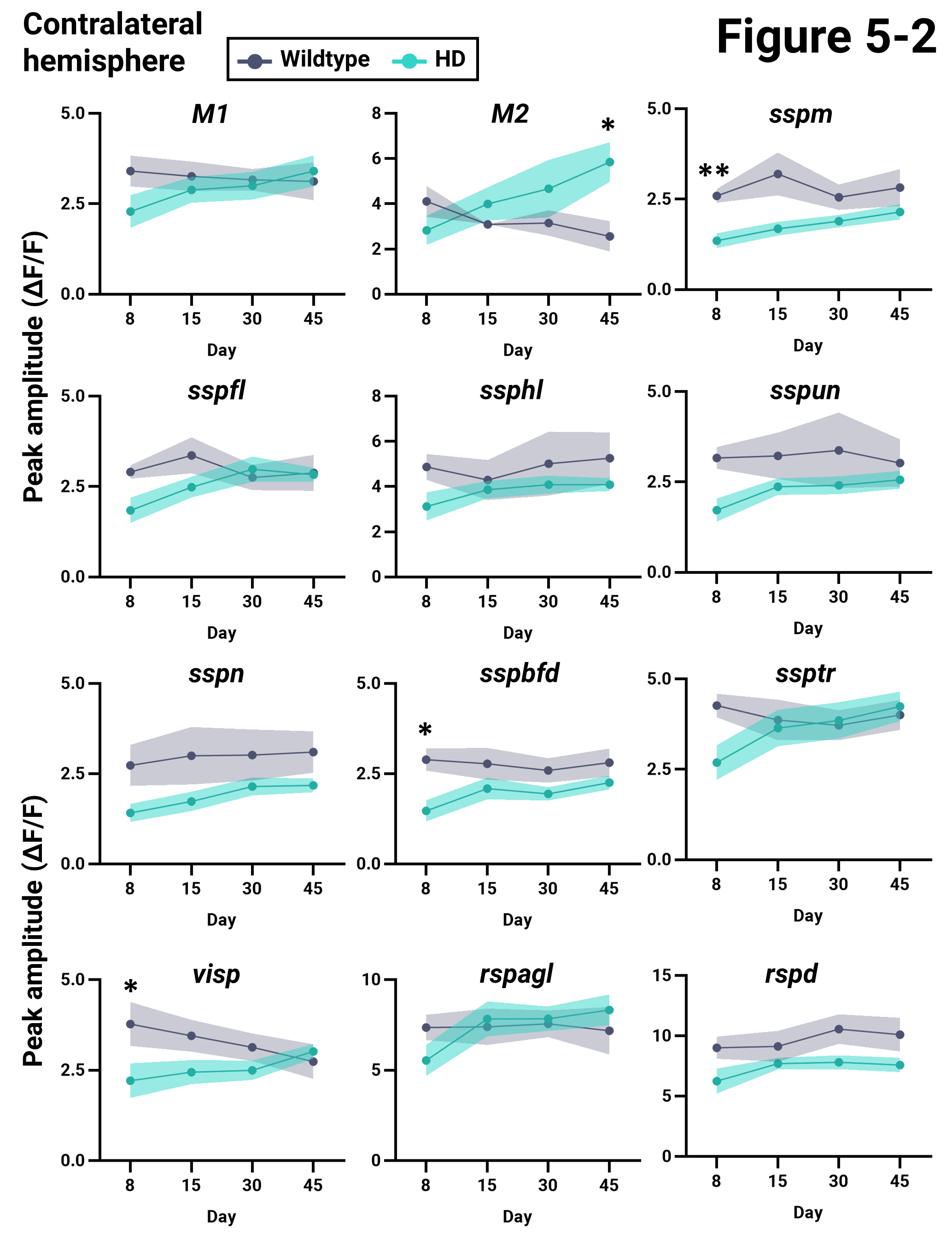

Supplement: Extended Data Figure 5-2 — Change in contralateral hemisphere ROI peak ΔF/F amplitude over time. Peak ΔF/F amplitude of ROIs in the contralateral hemisphere over time for WT (n = 4; gray) and HD (n = 6; teal) mice; **p < 0.01, *p < 0.05. Download Figure 5-2, TIF file. [file enu-eN-MNT-0452-22-s07.tif]

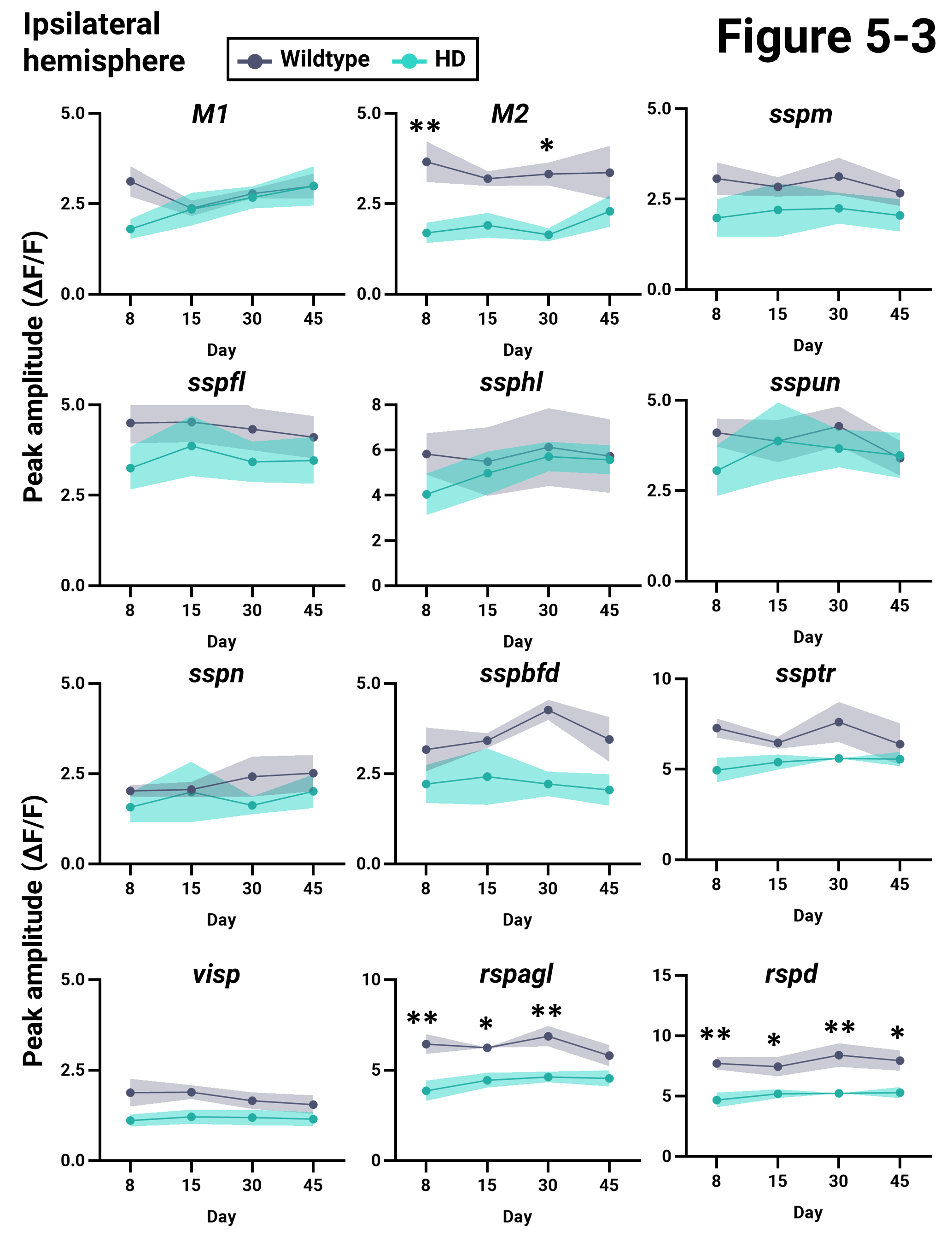

Supplement: Extended Data Figure 5-3 — Change in ipsilateral hemisphere ROI peak ΔF/F amplitude over time. Peak ΔF/F amplitude of ROIs in the ipsilateral hemisphere over time for WT (n = 4; gray) and HD (n = 6; teal) mice; **p < 0.01, *p < 0.05. Download Figure 5-3, TIF file. [file enu-eN-MNT-0452-22-s08.tif]

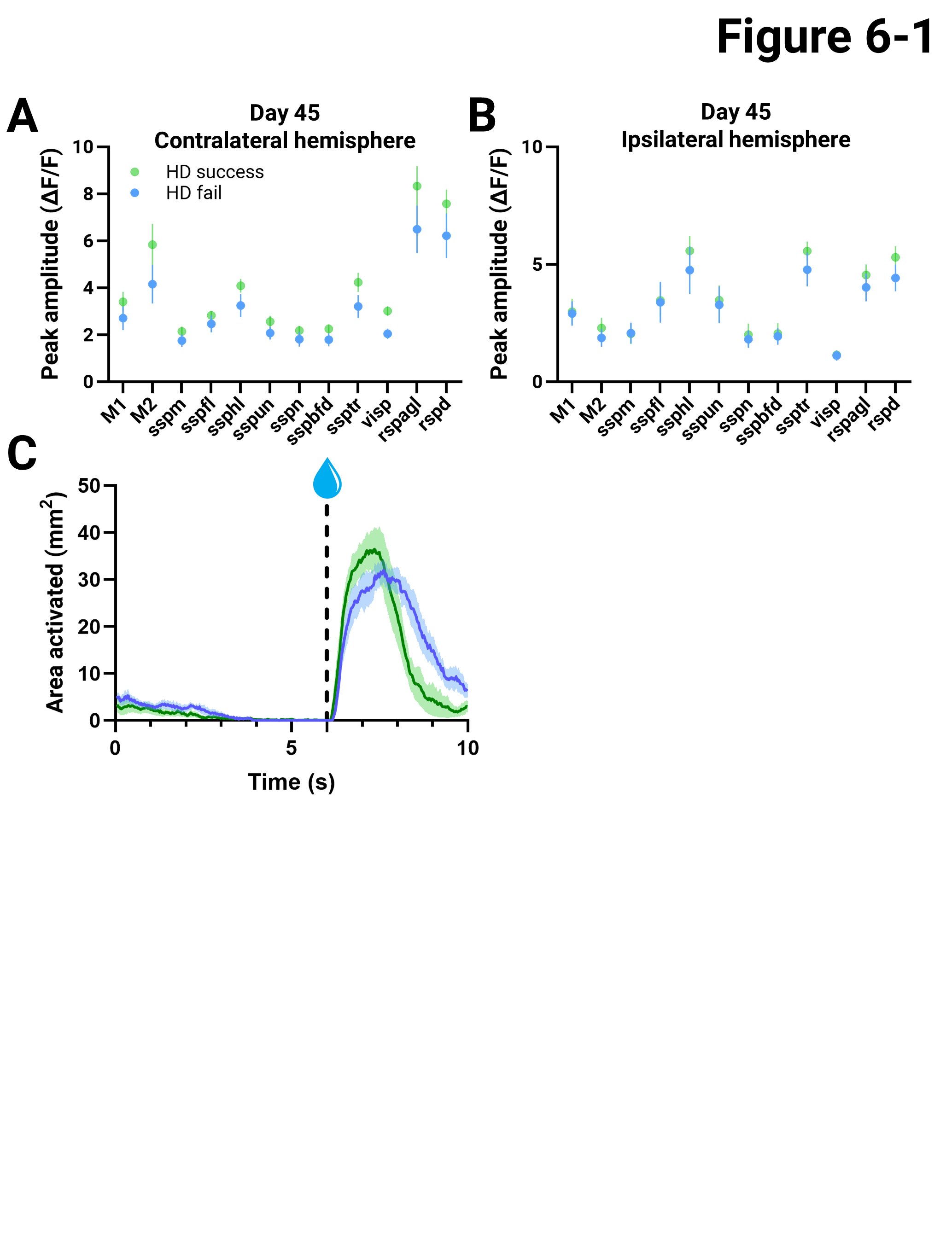

Supplement: Extended Data Figure 6-1 — Mesoscale GCaMP imaging of the cortex during success and fail trials performed by HD mice on Day 45. HD (n = 6) success and fail trials are denoted in green and blue, respectively. A, B, Peak amplitude of regions of interest in the contralateral (F(1,10) = 2.540, p = 0.1421, ANOVA; A) and ipsilateral (F(1,10) = 0.2573, p = 0.6230, ANOVA; B) hemisphere for different trial types. C, Area activated across the entire trial duration for successful and failed trials. The threshold was set at 4× SD (STD) of the baseline. No significance between trial types. Download Figure 6-1, TIF file. [file enu-eN-MNT-0452-22-s09.tif]
